# Co-Administration of Adjuvanted Recombinant *Ov*-103 and *Ov*-RAL-2 Vaccines Confer Protection against Natural Challenge in A Bovine *Onchocerca ochengi* Infection Model of Human Onchocerciasis

**DOI:** 10.3390/vaccines10060861

**Published:** 2022-05-27

**Authors:** Lisa Luu, Germanus S. Bah, Ndode Herman Okah-Nnane, Catherine S. Hartley, Alexandra F. Glover, Tessa R. Walsh, Lu-Yun Lian, Bin Zhan, Maria Elena Bottazzi, David Abraham, Nikolai Petrovsky, Nicolas Bayang, Bernard Tangwa, Rene Billingwe Ayiseh, Glory Enjong Mbah, David D. Ekale, Vincent N. Tanya, Sara Lustigman, Benjamin L. Makepeace, John Graham-Brown

**Affiliations:** 1 Institute of Infection, Veterinary & Ecological Sciences, University of Liverpool, Liverpool L3 5RF, UK; lisaluu@liverpool.ac.uk (L.L.); csguy@liverpool.ac.uk (C.S.H.); hlaglov3@liverpool.ac.uk (A.F.G.); hltwalsh@liverpool.ac.uk (T.R.W.); 2 L’Institut de Recherche Agricole Pour le Deéveloppement (IRAD), Yaoundé 2123, Cameroon; bahsohg2002@yahoo.com (G.S.B.); ndodeherman@gmail.com (N.H.O.-N.); bhnveto@yahoo.fe (N.B.); viban05viban@gmail.com (B.T.); dibongoekale@yahoo.fr (D.D.E.); vntanya@yahoo.com (V.N.T.); 3 Institute of Systems, Molecular and Integrative Biology, University of Liverpool, Liverpool L3 5RF, UK; lylian1@liverpool.ac.uk; 4 National School of Tropical Medicine, Baylor College of Medicine, Houston, TX 77030, USA; bzhan@bcm.edu (B.Z.); bottazzi@bcm.edu (M.E.B.); 5 Department of Microbiology and Immunology, Sidney Kimmel Medical College, Thomas Jefferson University, Philadelphia, PA 19107, USA; david.abraham@jefferson.edu; 6 Flinders Medical Centre, Adelaide 5042, Australia; nikolai.petrovsky@flinders.edu.au; 7 Biotechnology Unit, University of Buea, Buea P.O. Box 63, Cameroon; rbilingwe@yahoo.com; 8 Department of Biology, Higher Teacher Training College (HTTC), The University of Bamenda, Bambili P.O. Box 39, Cameroon; gmbah21@gmail.com; 9 Molecular Parasitology, Lindsley F. Kimball Research Institute, New York Blood Center, New York, NY 10065, USA

**Keywords:** river blindness, onchocerciasis, NTDs, vaccine, immunity, One Health

## Abstract

Onchocerciasis (river blindness), caused by the filarial nematode *Onchocerca volvulus*, is a neglected tropical disease mainly of sub-Saharan Africa. Worldwide, an estimated 20.9 million individuals live with infection and a further 205 million are at risk of disease. Current control methods rely on mass drug administration of ivermectin to kill microfilariae and inhibit female worm fecundity. The identification and development of efficacious vaccines as complementary preventive tools to support ongoing elimination efforts are therefore an important objective of onchocerciasis research. We evaluated the protective effects of co-administering leading *O. volvulus*-derived recombinant vaccine candidates (*Ov*-103 and *Ov*-RAL-2) with subsequent natural exposure to the closely related cattle parasite *Onchocerca ochengi*. Over a 24-month exposure period, vaccinated calves (*n* = 11) were shown to acquire infection and microfilaridermia at a significantly lower rate compared to unvaccinated control animals (*n* = 10). Furthermore, adult female worm burdens were negatively correlated with anti-*Ov*-103 and *Ov*-RAL-2 IgG1 and IgG2 responses. Peptide arrays identified several *Ov*-103 and *Ov*-RAL-2-specific epitopes homologous to those identified as human B-cell and helper T-cell epitope candidates and by naturally-infected human subjects in previous studies. Overall, this study demonstrates co-administration of *Ov*-103 and *Ov*-RAL-2 with Montanide™ ISA 206 VG is highly immunogenic in cattle, conferring partial protection against natural challenge with *O. ochengi*. The strong, antigen-specific IgG1 and IgG2 responses associated with vaccine-induced protection are highly suggestive of a mixed Th1/Th2 associated antibody responses. Collectively, this evidence suggests vaccine formulations for human onchocerciasis should aim to elicit similarly balanced Th1/Th2 immune responses.

## 1. Introduction

Human onchocerciasis (river blindness), a disease caused by the filarial nematode *Onchocerca volvulus*, is endemic to 27 countries across sub-Saharan Africa, alongside Venezuela, Brazil and Yemen, with an estimated 205 million people at risk from infection [1]. The Global Burden of Disease Study estimated 20.9 million *O. volvulus* infections worldwide, with around 14.6 million individuals affected by associated skin disease and 1.15 million individuals suffering from loss of vision [2]. 

Whilst long-term elimination programmes based on mass drug administration (MDA) of the microfilaricidal drug ivermectin have achieved elimination in Colombia, Ecuador, Mexico and Guatemala, progress has been much slower than anticipated elsewhere, particularly endemic regions of Africa. Thus, with an estimated 31% reduction in incidence observed across the continent from 1995 to 2015 [3], the African Programme for Onchocerciasis Control’s target of achieving elimination in 80% of endemic countries by 2025 will not be met [4]. Elimination efforts in Africa have been complicated by a number of factors, including the contra-indication of ivermectin MDA in areas co-endemic with *Loa loa* due to potentially severe adverse effects in loiasis patients [5]. Furthermore, reports of reduced duration of suppression of microfilarial burdens by ivermectin in some foci are concerning and have implications for continued reliance on MDA programmes to interrupt transmission long-term [6]. Epidemiological modelling suggests elimination efforts based exclusively on MDA will not achieve disease elimination [7], whilst the consensus amongst experts (~70%) is that additional measures are required to eliminate human onchocerciasis [8]. The recent licencing of the macrocyclic lactone moxidectin for use as an additional microfilaricidal treatment is promising [9,10,11], whilst efforts to identify and evaluate safe drugs targeting adult worms (macrofilaricides) are ongoing [12,13]. 

Vaccination has the potential to serve as an important complementary tool to aid disease elimination efforts through protecting from infection (prophylactic), reduction of disease severity (therapeutic) and reduction of transmission (anti-fecundity) [14,15]. The Onchocerciasis Vaccine for Africa (TOVA) consortium aims to develop a prophylactic vaccine based on recombinant subunit antigens to support MDA programs. In recent years, two recombinant antigen candidates, *Ov*-103 and *Ov*-RAL-2, have emerged as leading vaccine candidates, showing promising results both in vitro and in small animal experimental infection models, including heterogenous outbred populations against *O. volvulus*, as well as in a gerbil-*Brugia malayi* model using *B. malayi*-specific recombinant 103 and RAL-2 homologues [16,17,18,19,20]. Importantly, these studies have also determined key immunological mechanisms associated with vaccine-induced protection. Through the use of AID^-/-^ mice, it has been demonstrated that antigen-specific IgG responses are an essential component of a protective immune response through the facilitation of antibody-dependent cell-mediated cytotoxicity (ADCC) [17]. Further characterisation of these vaccine-induced immune responses in mice has identified a mixture of both Th1 and Th2 responses are associated with immunological protection [19]. Similarly, in a previous experimental vaccine trial in cattle using eight recombinant *O. ochengi*-derived antigen candidates (including *Oo*-RAL-2), a significant reduction of microfilaridermia in vaccinated versus control animals following natural exposure to *O. ochengi* was associated with antigen-specific IgG1 and IgG2 isotype responses [21]. Notably, in cattle, the IgG2 isotype is commonly associated with Th1-type immune responses and ADCC activity [22,23]. The importance of vaccine-induced ADCC as a protective mechanism against *Onchocerca* spp. is further supported by an in vitro study where human monospecific anti-*Ov*-103 and anti-*Ov*-RAL-2 antibodies were shown to inhibit moulting of *O. volvulus* L3 by 70–80% in the presence of naïve human monocytes [17]. Furthermore, it has been demonstrated that *Ov*-103 and *Ov*-RAL-2 induce protective immunity through a variety of immunological mechanisms, with a demonstrable synergistic protective effect when these antigens are co-administered, and efficacy across a wide range of host genetic diversity [16,17,19,20].

The bovine-*Onchocerca ochengi* natural transmission model has been used extensively for drug discovery, evaluation of diagnostics and vaccine trials [24]. *Onchocerca ochengi* is phylogenetically the closest known relative of *O. volvulus* and these parasites share near-identical life cycles, including the same vector species complex (*Simulium damnosum s.l.*). Accordingly, the protein sequences of *O. volvulus* and *O. ochengi* are 99.4% and 100% identical for *Ov*-103 and *Ov*-RAL-2, respectively. This system therefore presents a valuable opportunity to assess the efficacy of potential vaccine candidates in a field environment with natural exposure before proceeding to a phase 1 clinical trial in humans.

To inform ongoing vaccine development for human onchocerciasis, in the current study we evaluated the effects of immunisation with *Ov*-103 and *Ov*-RAL-2 on the immune responses and subsequent infection rates in cattle when naturally exposed to *O. ochengi*. Specifically, we conducted an initial immunogenicity trial to identify appropriate formulation in cattle by assessing three different adjuvants, aluminium oxide (Rehydragel® LV), a delta inulin plus CpG oligodendronucleotide (ODN)-based adjuvant (Advax^TM^-2), and a water-in-oil-in-water (w/o/w) emulsion (Montanide^TM^ ISA 206 VG). The most appropriate formulation (Montanide^TM^ ISA 206 VG) was then taken forward for use in a full vaccine efficacy trial. Here, we demonstrate that co-administration of *Ov*-103 and *Ov*-RAL-2 with Montanide™ ISA 206 VG is highly immunogenic, and confers partial protection in cattle against natural challenge with *O. ochengi* that is associated with a strong, mixed antigen-specific IgG1 and IgG2 response.

## 2. Materials and Methods

To ensure all observations and measurements were unbiased during the fieldwork phase of this study, researchers and technicians were blinded to the experimental group to which study animals were designated, with the exception of the individual responsible for both formulation and administration of the vaccine and control preparations.

### 2.1. Recombinant Antigen Production 

The recombinant *Ov*-103 protein was highly expressed in *Pichia pastoris* (PichiaPink^TM^ strain#1, Invitrogen) at the 10 L scale fermentation and purified by one-step cation ion exchange chromatography using HiTrap SP (GE Healthcare, Chicago, IL, USA). The recombinant *Ov*-RAL-2 protein was expressed in *E. coli* BL21(DE3) under induction of 1 mM IPTG. The expressed soluble recombinant *Ov*-RAL-2 with His-tag at C-terminus was purified with immobilised metal affinity chromatography (IMAC) with HisTrap™ FF (GE Healthcare, Chicago, IL, USA) followed by Q column polishing step to remove contaminated endotoxin [17,18]. The level of endotoxin in the final products of both recombinant proteins was less than 20 EU/mg (13.2–19.3 EU/mg). 

### 2.2. Calf Husbandry and Field Site

**Figure 1:**
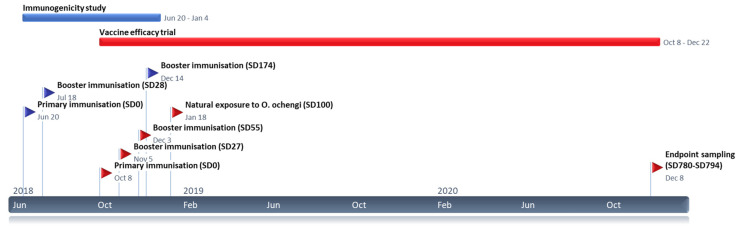
Timeline of critical events and durations of the vaccine immunogenicity study (**blue**) and vaccine efficacy trial (**red**).

Calf husbandry and experimental work was conducted at the Institut de Recherche Agricole pour le Développement (IRAD) field station at Wakwa (7.265° N, 13.548° E), near Ngaoundéré, Adamawa Region, Cameroon. Twelve, and 24 naïve calves were obtained for the immunogenicity trial and the main vaccine and exposure trial, respectively, from locations where *O. ochengi* transmission was absent. This was determined by a historic absence of disease and vector habitat at each purchase location, and the examination of adult cattle at these premises for evidence of *O. ochengi* nodules and *Onchocerca* spp. microfilaridermia by skin snips (Section 2.5). Upon arrival at the field station, all calves were placed and maintained in fly-proof housing with *ad libitum* access to water until natural exposure to *O. ochengi* was indicated (Figure 1). Prior to study commencement, all 24 calves enrolled in the vaccine efficacy trial were examined for evidence of infection by palpation for nodules and skin snips to identify microfilaridermia (Section 2.5). 

All calves were purchased and weaned at least 4 weeks in advance of study commencement, and aged 4–6 months at the point of primary immunisation. In addition to study-specific practices, general animal health and husbandry interventions were routinely implemented to help maintain animal health and welfare (Table S1). All animals were immunised against common endemic diseases (contagious bovine pleuropneumonia, lumpy skin disease, blackleg and pasteurellosis) prior to study commencement and boosted annually. All calves were manually de-ticked on a daily basis and tested routinely every 6 months for bovine tuberculosis (Bovigam^TM^, Prionics^TM^) and African animal trypanosomiasis (VerY Diag, Ceva Animal Health, Amersham, UK), with further monthly diagnostic assessment of calves for other haemoparasites (e.g., *Anaplasma* spp, *Babesia* spp etc.) through microscopy of blood smears, and hepatic (*Fasciola gigantica*) and gastro-intestinal parasites through standard coprological examination techniques for trematode and nematode eggs. Where treatment interventions were indicated, pharmaceutical products with known activity against *Simulium* and/or *O. ochengi*, namely acaricide and anthelmintic preparations containing macrocyclic lactones, synthetic pyrethroids etc, and tetracycline-based antibiotics, were strictly prohibited to avoid impacts on natural exposure to, and subsequent infection with, *O. ochengi* [25]. Typically, when identified, trypanosomiasis and other haemoparasites (e.g., babesiosis and anaplasmosis) were treated with diminazene aceturate, gastro-intestinal nematodes with albendazole, and *F. gigantica* with nitroxinil. To avoid any such interventions inadvertently impacting study results, and to safeguard animal health and welfare as much as possible, where treatment of these infections was indicated in one or more individuals, it was also applied prophylactically to all animals within the study group. Additional one-off supportive and/or therapeutic treatments for specific maladies identified over the study period (e.g., generalised trichophytosis) were applied only to affected individuals. All clinical diagnostic and treatment data was recorded for study auditing purposes.

### 2.3. Immunisation Strategy

Immunisation experiments in cattle with recombinant *Ov*-103 and *Ov*-RAL-2 occurred in two distinct phases (Figure 1). In all cases, vaccine preparations were formulated to a standardised antigen dose and administration protocol as described. At each immunisation timepoint, vaccinated animals received separate immunisations containing recombinant *Ov*-103 and *Ov*-RAL-2 antigens diluted in 1× Tris-buffered saline (TBS) plus adjuvant (see below) at a total volume of 2 mL per dose by contralateral intramuscular injection into the tensor fascia latae. Primary immunisations were administered at a dose of 500 µg for each antigen, with subsequent booster immunisations administered at a dose of 250 µg for each antigen.

#### 2.3.1. Immunogenicity Trial

To evaluate the immunogenicity of different vaccine formulations, twelve calves were sorted by age and sex, then randomly assigned to one of four equal groups (*n* = 3). Three groups were immunised as described above with one of three adjuvant formulations. These were 2% aluminium oxide (Al_2_O_3_) (Rehydragel® LV) mixed at a 1:28.5 adjuvant/antigen ratio) with a final concentration of 0.07% aluminium oxide; a delta inulin plus CpG oligodendronucleotide (ODN)-based adjuvant (Advax^TM^-2; 1:2.5 adjuvant/antigen ratio) with a final concentration of 50 mg delta inulin and 0.5 mg CpG per dose; or a water-in-oil-in-water (w/o/w) emulsion (Montanide^TM^ ISA 206 VG, Seppic; 1:1 adjuvant/antigen ratio). Alum-antigen preparations were prepared one day in advance to allow for antigen binding and stored at 4 °C overnight. Rehydragel®-antigen binding was then confirmed (>99%) by Bradford assay and SDS-PAGE analysis of vaccine supernatants following centrifugations at 10,000× *g* twice for 2 min. Both Advax-2 and Montanide ISA 206 VG formulations were prepared by vortex-mixing to emulsify the antigen-adjuvant components following manufacturer’s recommendations. The fourth experimental group received no immunisations, acting as an unvaccinated control group. Following primary immunisation on Study Day zero (“SD0”) (20 June 2018), an initial booster immunisation (250 µg per antigen in 2 mL) was administered at four weeks (SD28). To evaluate subsequent boosting effect, a further booster immunisation was administered at 6 months (SD174; Figure 1). 

#### 2.3.2. Vaccine Efficacy Trial

After identifying a suitable vaccine adjuvant formulation in the immunogenicity trial (see results), 24 calves were sorted by age and sex, and randomly assigned to one of two equal groups. The vaccine group were immunised with recombinant *Ov*-103 and *Ov*-RAL-2 antigens formulated with a water-in-oil-in-water (w/o/w) emulsion (Montanide^TM^ ISA 206 VG; 1:1 adjuvant/antigen ratio) as described above. The second experimental (control) group received two formulations containing TBS and adjuvant only (2 mL per dose). Following primary immunisations (SD0; 8 October 2018), two booster immunisations at four and eight weeks (SD27 and SD55; Figure 1) were administered in line with previous anti-*O. ochengi* vaccine efficacy trials in cattle [21]. Following each immunisation, calves were monitored continually for a period of 40 min for signs of adverse reactions.

### 2.4. Natural Exposure to O. ochengi

To determine the effect of immunisation on acquisition of *O. ochengi* infection, animals in the vaccine efficacy trial (*n* = 24) were continually exposed to natural challenge 6 weeks after their second booster immunisation (SD102; 18 January 2019) for a period of 24 months (680 days; Figure 1). This was achieved by transfer of animals by foot, with subsequent maintenance at pastures located adjacent to the Vina du Sud (7.215° N, 13.597° E), a site approximately 9 km from the IRAD field station with previously confirmed *O. ochengi* transmission [26,27]. Ongoing transmission rates were also monitored through regular entomological assessment (Section 2.7). To further increase definitive host density and boost *O. ochengi* transmission at this location, all animals from the immunogenicity trial (*n* = 12) and three adult cattle with confirmed patent *O. ochengi* infection were also transferred and maintained at the Vina du Sud alongside the vaccine efficacy trial animals. 

### 2.5. Routine Sampling and Clinical Observations

All calves were routinely examined and sampled weekly over the immunisation period (SD0–SD42 for the immunogenicity trial and SD0–SD91 for the vaccine efficacy trial) then, where possible, monthly over a total period of 196 days and 780 days for the immunogenicity and vaccine efficacy trials, respectively. Routine examination included a full clinical examination of each animal by a qualified veterinary surgeon.

Routine sampling included collection of serum and EDTA-treated whole blood by jugular venepuncture; the latter was used to determine total and differential leucocyte counts. Whole blood was diluted 1:20 in lysis buffer (0.9% NH_4_Cl) and total counts were obtained using an improved Neubauer haemacytometer. Differential counts were performed on thin blood smears through systematic observation of the feathered edge at 100× magnification under oil immersion following methanol fixation (5 min), Giemsa staining (1:7 dilution in distilled water for 90 min), and rinsing in PBS (pH 7.1). Total and differential counts were used to calculate total circulating volume of each cell phenotype for subsequent statistical analysis. EDTA-treated whole blood was also used for isolation of peripheral blood mononuclear cells (PBMCs) for subsequent ex vivo culture and proliferation assays. Clotted blood was centrifuged to separate serum and stored at −80 °C. To meet the importation requirements issued by the UK government’s Animal and Plant Health Agency (APHA; ITIMP 18.0881), sera were heat-treated at 56 °C for 30 min prior to shipment to the UK.

For the vaccine efficacy trial, from 6 months post-exposure onwards, monthly clinical examinations included palpation and inspection of the dermis of each animal’s ventrum for *O. ochengi* nodules and their locations were marked on a “hide map”. Skin snips were performed in triplicate to detect *Onchocerca* spp. microfilarial burdens by microscopy following incubation and collagenase digestion as described previously [28]. To achieve this, each animal was placed in lateral recumbency for a period not exceeding 30 min.

### 2.6. End-Point Sampling

At the conclusion of the 24-month natural exposure period of the vaccine efficacy trial, up to 30 female worm nodules were surgically excised from all remaining animals (*n* = 21) by a qualified veterinary surgeon under local anaesthesia. Animals were placed and restrained in lateral recumbency for a period no longer than 30 min, and ≤10 nodules were excised per sampling, with a minimum of 7 days between surgeries. Upon excision, all nodules were trimmed of excess host connective tissue to aid with definitive identification as *O. ochengi* nodules. The long and short axis diameters were measured using vernier callipers and the nodules were weighed. For animals with nodule burdens > 30, final burdens were determined through a combination of these surgical excisions and palpation of any remaining nodules.

In addition, up to three nodules per animal were randomly selected from each animal for further analyses through microscopic dissection and examination in PBS to determine female worm viability, motility, age, fecundity; male worm numbers, and motility [28]. Intact males and dissected female heads were removed to further evaluate viability by 3-(4,5-dimethylthiazol-2-yl)-2,5-diphenyltetrazolium bromide (MTT) assay for metabolic activity [28]; whilst female worm bodies were submitted for assessment of overall reproductive status through quantification of normal and degenerate oocytes, embryonic stages, and intrauterine Mf by embryogram as described previously [28]. 

### 2.7. Entomological Analyses and Transmission Potential

To confirm the presence of circulating *O. ochengi* parasites at the Vina du Sud transmission site, quantify vector biting rates and *O. ochengi* transmission potential, fly catching was conducted for *Simulium* spp. as described previously [26]. Briefly, a study calf was tethered for a period of up to three hours within the transmission site and used as bait to attract *Simulium* spp. Flies were collected manually from the ventrum and legs, typically for 7–9 h per day from 7:00 to 18:00 h on a weekly (to fortnightly) basis. To account for, and minimise, the impact of variation in attractiveness of individual cattle on *Simulium* spp. biting rates, sampling was performed repeatedly on four randomly pre-selected study animals in rotation by week and sampling period (morning or afternoon). Flies were collected, with catch numbers recorded on an hourly basis, and stored at −20 °C until dissection. 

Up to 200 female *Simulium damnosum s.l.* specimens per timepoint were dissected; fly abdomens were dissected to distinguish parous from nulliparous flies through examination of ovarian and malphigian tubule texture and morphology [29,30]. Parous flies were then examined for the presence of *Onchocerca* spp. larvae in the head, thorax and abdomen with the number of L1, L2 and L3 recorded for each fly. *O. ochengi* filariae were identified by morphological parameters including body length, and the shape of the anterior and posterior ends as previously described [31,32,33]. 

Data spanning the entire exposure period (November 2018 to November 2020) was used to calculate biting rates, infection rates and transmission potentials as described previously [29]. Briefly, monthly biting rate (MBR) was calculated from the overall daily catch rate for each month without corrections for lost catching hours due to external factors (e.g., heavy rain). Monthly infection rate (MIR) was calculated as the total number of infective *Onchocerca* spp. L3 identified divided by the number of dissected flies, with monthly transmission potential (MTP) then calculated by multiplying MBR and MIR for each month. To allow comparison of natural exposure and infectivity to previous studies, annual biting rates (ABR) and transmission potential (ATP) were also calculated for the complete hydrological year spanned by the study period (April 2019 to March 2020).

### 2.8. Measuring Anti-Ov-103 and Ov-Ral-2 IgG Responses

Serum samples were assayed to measure total IgG ELISA and its constituent isotypes IgG1 and IgG2 ELISAs as follows. Immulon™ 2HB 96-well plates (Nunc) were coated with either *Ov*-103 or *Ov*-RAL-2 at a concentration of 1 µg/mL in 0.1 M carbonate buffer (pH 9.6) and incubated overnight at 4 °C. Plates were washed with 0.1% Tween-20 in PBS (wash buffer) between each incubation step with two cycles of two rapid (no incubation) and one long (5 min) wash. Plates were blocked with a 2% Marvel® milk powder in wash buffer (blocking buffer), 200 µL/well, overnight at 4 °C. All subsequent steps were performed at room temperature. Individual sera were diluted in blocking buffer and assayed in duplicate and incubated for 2 h. To assess the kinetics of serum antibody responses, serum samples were diluted and assayed at 1:800. Specifically, for total IgG, all serum collected between SD0 to SD196 (immunogenicity trial) or SD780 (vaccine efficacy trial) was assayed, and for IgG1 and IgG2 isotypes, all serum collected between SD0 to SD42 for the immunogenicity trial. Additionally, to determine end-point antibody titrations, serum from selected timepoints (immunogenicity study: SD189; vaccine efficacy study SD0, SD27, SD55, SD62, SD89, SD215, SD636 and SD780) were assayed as threefold serial dilutions (1:800 to 1:1,614,600) for total IgG, IgG1 and IgG2. Secondary detection antibodies were diluted in blocking buffer and incubated for 2 h, 100 µL/well, as follows: HRP-conjugated sheep anti-bovine IgG (polyclonal IgG; Bio-Rad AAI23P; 1:120,000), sheep anti-bovine IgG1 (polyclonal IgG; Bio-Rad AAI21P; 1:100,000) and sheep anti-bovine IgG2 (polyclonal IgG; Bio-Rad AAI22P; 1:100,000). The detection substrate 3,3′,5,5′-tetramethylbenzidine (TMB; Cheshire Bioscience) was incubated, 100µL/well, in the dark for 20 min, with development stopped by addition of 0.5M HCl. Optical densities (OD) were read at 450 nm using a Tecan Infinite F50 microplate reader with Magellan for F50 analysis software (Tecan).

For antibody kinetics analysis, sample OD data was normalised across plates through expression as a (blank-reduced) ratio versus a high-responding sample identified in the initial 6-week immunisation period (animal 2909, SD35) and run across all subsequent plates. A nominal antibody positive/negative “threshold” value for longitudinal (1:800) serum antibody ELISA data was calculated as the mean OD ratio plus 3 SD for all negative sample wells. Serum samples registering OD ratios greater than this value are described as having detectable levels of antigen-specific antibody response. 

End-point titrations for total IgG, IgG1 and IgG2 were calculated from blank-reduced OD values of serial dilutions (1:800 to 1:1,614,600). OD data from each respective individual, timepoint and isotype was fitted to a non-linear least squares exponential decay model and compared to a similarly constructed negative cut-off curve (upper 95% confidence interval plus 2 SD), which was fitted to blank-reduced serial diluted OD values from all animals measured at SD0 in R-studio [34]. End-point titres for each animal, IgG isotype and timepoint were defined as the dilution at which fitted OD values dropped below the fitted end-point cut-off curve.

### 2.9. IgG Purification and Epitope Mapping

To further investigate vaccine induced antibody responses, IgG was purified from a subset of sera samples (2917, 2938, 2944, 2946) at peak antibody titre, one-week post-secondary booster (SD62), and a single animal (2938) at four months (SD215) using GraviTrap™ Protein G columns (GE Healthcare) according to the manufacturer’s protocol at room temperature. Columns were equilibrated with binding buffer (20 mM sodium phosphate, pH 7.0) and aliquots of 750 µL sera were combined with 250 µL binding buffer, added to the column and washed with binding buffer. Purified IgG was eluted in 3 mL of 0.1 M glycine-HCl (pH 2.7) and 60 µL–200 μL/mL of neutralising buffer (1M Tris-HCl, pH 9.0) was added. pH was checked using pH indicator paper to confirm neutralisation to ~pH 7.0. Purified IgG was stored at 4 °C prior to shipment.

Bovine IgG epitope mapping was performed with a peptide microarray (JPT Peptide Technologies GmbH) containing a total of 76 peptides representing overlapping sequences derived from *Ov*-RAL-2 or *Ov*-103. Peptides were synthesized and immobilized on peptide microarray slides as described previously [35]. In brief, the peptides were synthesized using SPOT synthesis, cleaved from the solid support and chemoselectively immobilized on functionalized glass slides. Each peptide was deposited on the microarray in triplicate. The peptide microarrays were incubated with bovine IgG samples (1:200 dilution) in a 96-well microarray incubation chamber for one hour at 30 °C, followed by incubation with either sheep anti-bovine IgG1 (1:5000 dilution, Bio-Rad Laboratories, AAI21B), or sheep anti-bovine IgG2 (1:1000 dilution, Bio-Rad Laboratories, AAI22F) detection antibodies. Subsequently, antibodies were incubated for detection with 0.1 μg/mL fluorescently labelled (Cy^TM^5) streptavidin (Jackson Immunoresearch, 016-170-084) or with fluorescently labelled (Cy^TM^5) anti-sheep IgG (applied dilution 1:1000, Jackson Immunoresearch, 713-175-147). Washing steps were performed after each incubation step with 0.1% Tween-20 in 1x TBS. After the final incubation step, the microarrays were washed and dried. Each microarray slide was scanned using a GenePix Scanner 4300 SL50 (Molecular Devices, Pixel size: 10 μm). Signal intensities were evaluated using GenePix Pro 7.0 analysis software (Molecular Devices). For each peptide, the MMC2 value of the three triplicates was calculated. The MMC2 value was equal to the mean value of all three instances on the microarray except when the coefficient of variation (CV)–standard-deviation divided by the mean value–was larger than 0.5. In this case the mean of the two values closest to each other (MC2) was assigned to MMC2. Further data analyses and generation of the heatmaps were performed using the statistical computing and graphics software R (Version 4.1.1) [34].

### 2.10. Protein Structure Prediction

The structures of *Ov*-103 and *Ov*-RAL-2 were predicted using AlphaFold2 (AF2) [36] and RoseTTAFold [37]. In addition, the Alphafold predicted structures of both proteins are also available in the Uniprot Knowledgebase (*Ov*-103 is UniProtKB accession number Q99099, and *Ov*-RAL2 is UniProtKB accession number P36991). Very high to high per-residue confidence scores (pLDDT) in AlphaFold2 were obtained for residues ranging from G24 to LL132 for *Ov*-103, and R36 to L160 for *Ov*-RAL-2; it is likely that the regions with low pLDDT are unstructured in solution. Modelling by RoseTTAFold was performed using the Robetta server (https://robetta.bakerlab.org/, accessed on 7 March 2022). The RoseTTAFold structures of *Ov*-RAL-2 were similar to the AF2 structures. However, for *Ov*-RAL-2, although both AF2 and RF predicted a predominantly helical structure, with good structural alignments in some parts of the polypeptide chain, there were variations in other parts. AF2 structures were used for mapping the epitopes onto structures using Pymol (The PyMOL Molecular Graphics System, Schrödinger, LLC, New York, NY, USA)

### 2.11. PBMC Proliferation Assays

Following collection of EDTA-treated whole blood, PBMCs were isolated and purified as previously described [38]. Following purification, PBMCs were maintained in RPMI (w/o L-glutamine and phenol red, Sigma) supplemented with 10% foetal calf serum, L-glutamine (0.3 g/L) and penicillin and streptomycin (100 U/mL, 100 µg/mL) plated out in U-bottomed 96-well culture plates at 2 × 10^5^ cells (200 µL) per well, then stimulated over a 48-hour incubation period at 39 °C in a 5% CO_2_ environment (BD GasPak^TM^ EZ CO_2_ pouch system). All culture conditions were performed in triplicate and included unstimulated medium control, Concanavalin A mitogen (5 µg/mL), and *Ov*-103 and *Ov*-RAL-2 (both 10 µg/mL). To determine metabolic activity, 100 µg MTT (15M stock solution in PBS) was added to each culture well for the final four hours of incubation, after which culture medium was removed and the resultant formazan salt solubilised through overnight incubation in DMSO. The OD was measured at 595 nm on a plate reader (Labtech LT-4500) and antigen-stimulated culture proliferation was expressed as a stimulation index calculated by dividing mean culture stimulate OD by the corresponding medium control OD value.

### 2.12. Statistical Data Analysis

For the immunogenicity trial, due to the small group sizes and short sampling period, simple descriptive data analysis was performed *in lieu* of formal statistical testing. 

For the vaccine efficacy trial, formal statistical analysis was performed through multivariable linear mixed-effects models with residual maximum likelihood variance estimates in the “nlme” package within R-studio [34,39]. This analysis was divided into four distinct stages. In the three analyses involving longitudinal data (below), individual animal identification was included as a random-effect explanatory variable to account for individual animal heterogeneity on responsiveness to immunisation and subsequent natural exposure, in a dataset composed of repeated observations from these same individuals on multiple occasions.

#### 2.12.1. Immunisation Period

Data from SD0 (pre-immunisation) to SD83 (4 weeks post second booster) was analysed to determine the effect of immunisation status on immunological parameters modelled as response variables (Y). Specifically, anti-*Ov*-103 and anti-*Ov*-RAL-2 total serum IgG kinetics data, log(+1)-transformed IgG1 and IgG2 end-point titres, and total and differential (lymphocytes, monocytes and eosinophils) peripheral blood leucocyte counts were modelled independently to one another as response variables against multiple fixed-effect explanatory variables (x). For this analysis, fixed-effect explanatory variables comprised study day (time) to account for general changes over time independent of vaccination status, animal sex since previous studies have identified sex-related differences in immune and infection kinetics in the bovine *O. ochengi* infection model [40], and animal vaccination status as an interaction term with Study Day (time) to examine how immune response variables in vaccinated versus control group animals varied over the immunisation period. 

#### 2.12.2. Exposure Period

Data from SD89 (pre-exposure) to SD780 (24 months post exposure) was analysed to determine the effect of natural exposure to *O. ochengi* infection on both observable immunological and *O. ochengi* infection status parameters modelled as response variables (Y). Specifically, anti-*Ov*-103 and anti-*Ov*-RAL-2 total serum IgG kinetics data, log(+1)-transformed IgG1 and IgG2 end-point titres and total and differential (lymphocytes, monocytes and eosinophils) peripheral blood leucocyte counts, *O. ochengi* nodule counts and microfilarial burdens, were modelled independently to one another as response variables against multiple fixed-effect explanatory variables (x). For this analysis, fixed-effect explanatory variables comprised Study Day (time) to account for general changes over time independent of vaccination status, animal sex, and animal vaccination status as an interaction term with Study Day (time) to examine how immune and infection response variables in vaccinated versus control group animals varied over the exposure period. Additionally, for analysis of immunological parameters, vaccination status was also included as a separate explanatory variable, since vaccinated and control animals were expected to be immunologically distinct groups at the start of the exposure period.

#### 2.12.3. Assessing Immunological Correlates of Vaccine-Associated Protection

Where data analysis from the exposure period identified statistically significant immunological changes between immunised and control groups, these immunological parameters were incorporated into a further analysis of *O. ochengi* infection status in vaccinated calves as explanatory variables. Specifically, *O. ochengi* nodule counts and microfilarial burdens were modelled independently to one another as response variables (Y) against multiple fixed-effect explanatory variables (x). For this analysis, fixed-effect explanatory variables comprised animal sex, and the immunological variable of interest (e.g., anti-*Ov*-103 IgG) as both an independent explanatory variable, and as an interaction term with Study Day (time) to examine how immune responses correlated with acquisition of *O. ochengi* infection once immunological changes over time had been taken into account.

#### 2.12.4. Final Parasitological Burden

To determine the effect of immunisation status on final parasitological burden following a 22-month exposure period, detailed parasitological data collected at the final sampling were modelled as response variables (Y). Specifically, nodule age, average diameter, weight, number of male worms per nodule, motility, viability (as assessed by MTT assay) and fecundity, including specific and degenerate embryonic stages as measured by embryogram. Response variables, were modelled independently to one another against multiple fixed-effect explanatory variables (x). For this analysis, fixed-effect explanatory variables comprised animal sex and vaccination status. Where response variables included data from both male and female worms (e.g., motility), worm sex was included as an additional explanatory variable to account for the inherent differences in sex phenotype within *O. ochengi*.

## 3. Results

Thirty-six naïve calves aged 4–6 months were recruited for the immunogenicity trial (*n* = 12) and vaccine efficacy trial (*n* = 24). Prior to study commencement, no *O. ochengi* infection was detected in any calves, or their mothers, indicating these were an immunologically naïve cohort. Immunogenicity and vaccine efficacy trials were conducted as described (Figure 1 and Methods). No major adverse reactions were observed in calves following immunisation with any *Ov*-103 and *Ov*-RAL-2 vaccine candidate formulations and clinical parameters remained within their normal reference ranges throughout the study period. Over the total study period, five calves died as a result of health complications unrelated to the vaccine study (see Calf health).

### 3.1. Ov-103 and Ov-RAL-2 Immunisation in Cattle with Montanide^TM^ ISA 206VG Induces Pronounced, Rapid and Sustained Seroconversion

To identify a suitable vaccine formulation for the main vaccine efficacy trial, we conducted an immunogenicity trial using our candidate antigens in combination with one of three different adjuvants. 

**Figure 2:**
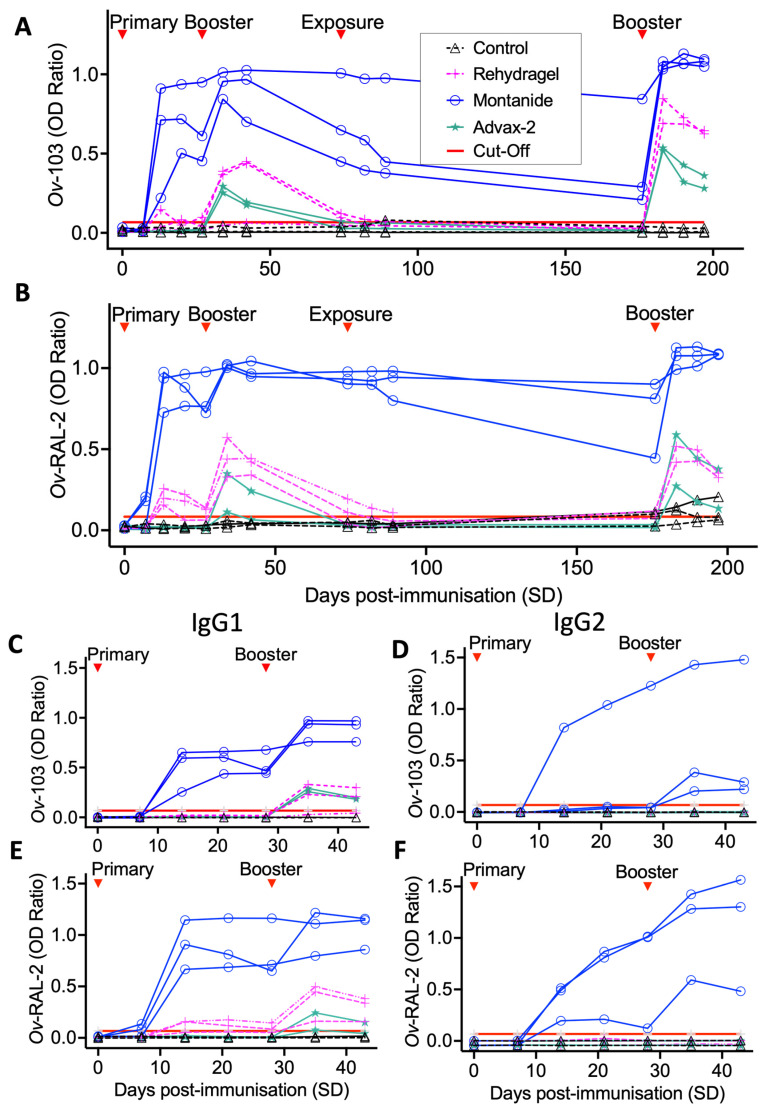
Timeseries of *Ov*-103 and *Ov*-RAL2-specific serum IgG responses for three different vaccine formulations and unvaccinated control animals assessed by ELISA (1:800 dilution) from pre-study immunogenicity trial. Relative timing of primary, booster immunisations and natural exposure are denoted for (**A**) anti-*Ov*-103 total IgG (**B**) anti-*Ov*-RAL-2 total IgG (**C**) anti-*Ov*-103 IgG1 (**D**) anti-*Ov*-103 IgG2 (**E**) anti-*Ov*-RAL-2 IgG1 (**F**) anti-O*v*-RAL-2 IgG2. Solid red line denotes each assay’s detection threshold for serum IgG.

All three vaccine formulations used in the immunogenicity trial groups (Montanide^TM^ ISA 206 VG, Rehydragel® and Advax-2^TM^ adjuvants) elicited elevations of *Ov*-103 and *Ov*-RAL-2-specific serum IgG compared to control animals (Figure 2). Overall, calves immunised with Montanide-adjuvanted formulations produced and sustained the highest levels of antigen-specific serum IgG responses. Elevations in anti-*Ov*-RAL-2 total IgG were observed in two Montanide group animals from SD7, with elevations in serum IgG responses to both antigens from SD14 onwards. These antibody responses were composed of both IgG1 and IgG2 isotypes to both *Ov*-103 and *Ov*-RAL-2, with IgG1 responses observed following primary immunisation and IgG2 responses following a booster at SD28 (Figure 2C–F). Conversely, Rehydragel and Advax-2-adjuvanted formulations elicited less marked IgG responses characterised exclusively as IgG1 isotype that were slower in onset and shorter in duration. Detectable levels of antigen-specific serum IgG were present in Rehydragel group animals from SD14 onwards, whilst IgG responses were only observed in Advax-2 animals at SD35 following the first booster (SD28) immunisation (Figure 2A,B). Total serum IgG remained low in unvaccinated control group animals, although a small increase in anti-*Ov*-RAL-2 IgG was observed following natural exposure (Figure 2B). 

In the Montanide group, serum IgG waned over time but remained well above baseline for both anti-*Ov*-103 and *Ov*-RAL-2 until the secondary booster (SD174), which elicited a strong and immediate serum IgG response (Figure 2A,B). A marked increase in IgG production was also induced in Rehydragel and Advax-2 group animals following the second booster immunisation, resulting in rapid increases of anti-*Ov*-103 and *Ov*-RAL-2 serum IgG which then progressively waned over subsequent sampling timepoints.

Observed differences in antibody responses between immunisation groups were further elucidated by end-point serum IgG, IgG1 and IgG2 isotype titrations performed at SD188, following a second booster immunisation (SD174). These showed total serum IgG and IgG1 responses in all immunised calves to be increased above those of control animals by several orders of magnitude, with Montanide group animals having end-point titres approximately 10–100-fold higher than those of Rehydragel and Advax-2 group animals. IgG2 responses were totally absent in Rehydragel and Advax-2 groups at this time, but detectable at high titres in all Montanide group calves (Table 1). In the Montanide group, serum IgG titres against *Ov*-RAL-2 were consistently higher than *Ov*-103.

### 3.2. Ov-103 and Ov-RAL-2 Immunisation in Cattle Is Associated with Reduced O. ochengi Female and Microfilarial Worm Burdens following Natural Exposure

Having identified Montanide™ ISA 206 VG adjuvant as a highly immunogenic vaccine formulation, we proceeded to evaluate whether this conferred protection against subsequent natural exposure to *O. ochengi* in a full vaccine efficacy trial. Following primary immunisation (SD0) and two boosters at 4-week intervals (SD27 and 55), animals were naturally exposed to *O. ochengi* (SD100) for a total of 24 months (SD780). Ongoing transmission was confirmed by regular surveillance of *O. ochengi* infection in boophilic blackflies sampled from bait cattle at the field site, which showed annual biting rates and transmission potential for the complete hydrological year (April 2019 to March 2020) calculated as 106,390 flies per cow and 7857 *O. ochengi* L3, respectively (Figure S1), which is comparable to previous observations at the Vina du Sud transmission site [26].

**Figure 3:**
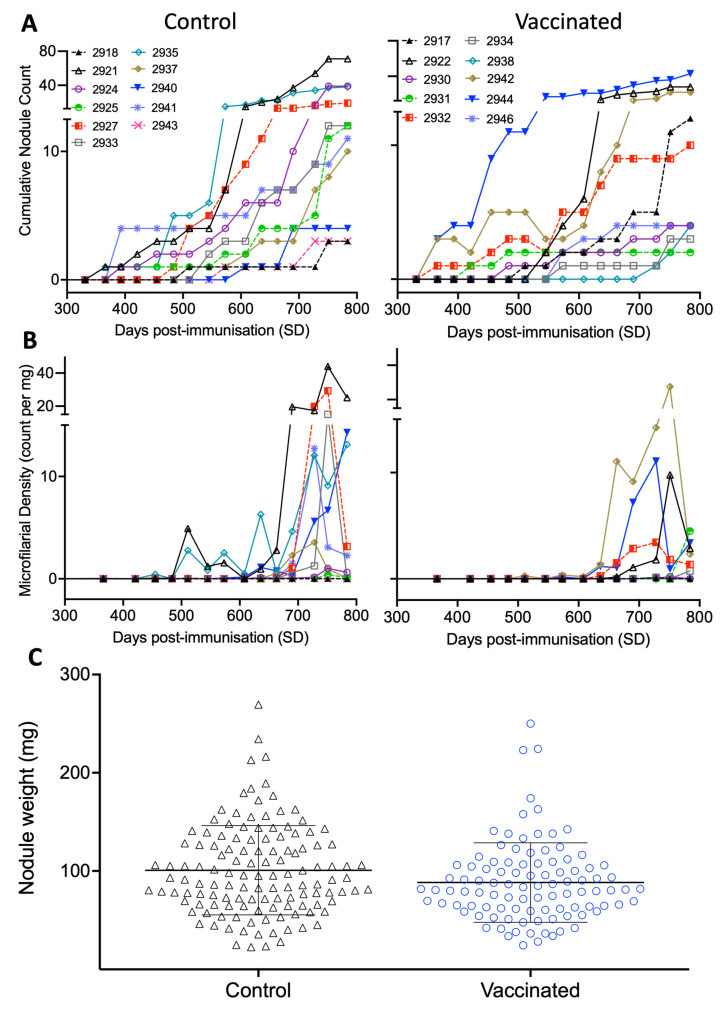
Parasitological burdens and in infection kinetics in main study animals resulting following natural exposure to *O. ochengi* (SD100). (**A**) Female worm burdens identified by manual palpation and confirmed at end-point sampling and (**B**) *Onchocerca* spp. microfilarial burdens within dermis identified through skin snips. (**C**) Weight of nodules (mg) excised from vaccine efficacy trial animals at endpoint. Bars denote mean with standard deviation.

Parasitological parameters were continually assessed in cattle to monitor for *O. ochengi* infection status and kinetics of acquisition, with animals observed to progressively accumulate *O. ochengi* burdens over the exposure period (Figure 3). Nodules were first observed at SD366 in two control and four vaccinated animals, nine months after initial exposure to *O. ochengi* (Figure 3A). *Onchocerca* spp. microfilaridermia was first identified at SD455 in one control animal (2935), then at SD511 in a vaccinated animal, at 12- and 14-months post exposure, respectively (Figure 3B). 

By the final sampling (SD780), mean female worm burdens and microfilarial (Mf) skin densities were 35% and 70% lower in vaccinated versus control animals, respectively. At this time, all calves (*n* = 21) had acquired nodules, with microfilaridermia identified in ten of the eleven control group animals, and nine of the ten vaccine group animals. These findings are summarised in combination with further detailed parasitological examination of male worm burdens, ages, viability, motility, fecundity and reproductive status in Table 2.

Multi-variable linear mixed-effect regression analysis from SD89–SD780 (including a pre-exposure reference timepoint) demonstrated that overall, both nodule and microfilarial burdens increased over the exposure period (identified through Time term; *p* < 0.001 in all cases; Table 3). However, rate of acquisition of both nodules and microfilaridermia over this period was significantly lower in vaccinated animals compared to the control group animals (identified through the Vaccination: Time interaction term; *p* = 0.0043 and 0.0085, respectively; Table 3). In combination, the fitted coefficients for the Time and Vaccination: Time explanatory variables indicated that: (1) Overall mean monthly increase (rate of acquisition) in control animals was 0.72 nodules and 0.24 Mf/mg (2) Mean monthly increase (rate of acquisition) in vaccinated animals was 0.53 nodules and 0.15 Mf/mg (3) Over the entire study period, there was a 26% and 39% reduction in the rate of acquisition of both nodules and microfilaridermia, respectively. Additionally, multi-variable linear mixed-effect regression analysis of end-point sampling data identified a statistically significant decrease in female worm nodule mass related to vaccination status (*p* < 0.05, Table S2; Figure 3C).

### 3.3. Vaccine-Induced Ov-103 and Ov-RAL-2-Specific Serum IgG Responses Are Associated with Reduced Adult O. ochengi Burdens following Natural Exposure

**Figure 4:**
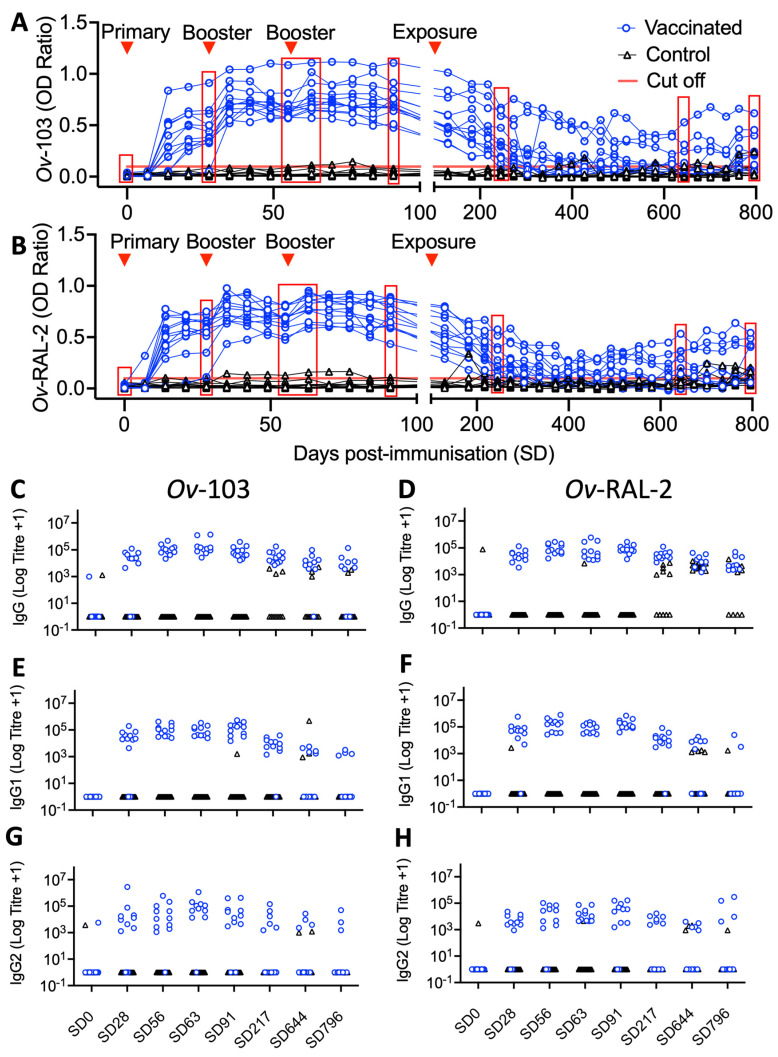
Timeseries of (**A**) anti-*Ov*-103 and (**B**) anti-*Ov*-RAL-2-specific serum IgG responses for vaccine efficacy trial animals by ELISA (1:800 dilution). Relative timing of primary, booster immunisations and natural exposure are denoted. Solid red line denotes each assay’s detection threshold for serum IgG. Highlighted timepoints (red boxes) denote those taken for IgG and isotype end-point serum antibody titrations (**C**–**H**), namely pre-immunisation (SD0), 4 weeks post primary immunization (SD28), 4 weeks post first booster immunization (SD56), 1 week post second booster immunization (SD63), 1 week pre-natural exposure (SD91), 4 months post-exposure (SD217), 18 months post-exposure (SD644), Final sampling (SD796).

For the vaccine efficacy trial, humoral immune responses to immunisation were continually monitored over both the immunisation and exposure periods. Similar to the immunogenicity trial, levels of both *Ov*-103 and *Ov*-RAL-2 antigen-specific serum IgG was detectable in all animals in the vaccinated group (Figure 4A,B). End-point antibody titrations from key timepoints, specifically pre-immunisation (SD0), first booster (SD27), pre-second (SD55) and 1-week post-booster (SD62), pre-exposure (SD89), 4 and 18 months post-exposure (SD215 and SD636, respectively) and end-point sampling (24 months post exposure; SD780), confirmed the marked but waning vaccine-induced antigen-specific IgG responses were composed of both IgG1 and IgG2 isotype antibodies (Figure 4C–H). 

During the immunisation period (SD0–SD83), antigen-specific IgG was detected after SD14 and was further stimulated following boosters at SD27 and, to a lesser extent, SD55 (Figure 4). Multi-variable linear mixed-effect regression analysis of the immunisation period identified this vaccine-induced antibody response to be statistically significant, with antigen-specific (anti-*Ov*-103 and *Ov*-RAL-2) total IgG OD ratios and logged end-point IgG1 and IgG2 titres all shown to increase over the immunisation period compared to control animals, where no such increases were seen (identified through the Vaccination:Time interaction term; *p* < 0.001 in all cases; Table 4). 

During the exposure period (SD102–SD780), antigen-specific IgG responses declined but overall remained elevated compared to control group animal responses up to SD215, after which six of 12 immunised animals maintained detectable levels of anti-*Ov*-103 and *Ov*-RAL-2 serum IgG throughout the majority of the remaining study period (565 days). Multi-variable linear mixed-effect regression analysis of the exposure period (SD89–SD780; includes pre-exposure reference timepoint) confirmed a statistically significant elevation in antigen-specific (anti-*Ov*-103 and *Ov*-RAL-2) total IgG OD ratios and logged end-point IgG1 and IgG2 titres in vaccinated calves compared to control animals (identified through Vaccination term; *p* < 0.001 in all cases; Table 5), but that these vaccine-induced antibody responses waned significantly over the exposure period (identified through Vaccination:Time interaction term; *p* < 0.001 in all cases; Table 5).

Following natural exposure, anti-*Ov*-103 and *Ov*-RAL-2 serum IgG responses (IgG1 and IgG2 isotypes) were detected in sera from control animals periodically from SD366 onwards (Figure 4C–H). Where vaccination status was accounted for through other explanatory variables, multi-variable linear mixed-effect regression analysis also indicated a statistically significant increase in logged anti-*Ov*-RAL-2 IgG1 and IgG2 antibody titres over the exposure period (Identified through Time term; *p* = 0.004 and 0.02, respectively; Table 5). 

When immunological antigen-specific (anti-*Ov*-103 and *Ov*-RAL-2) total IgG OD ratios and logged endpoint IgG1 and IgG2 titres were included as explanatory variables in multi-variable linear mixed-effect regression analysis examining *O. ochengi* infection kinetics in vaccinated animals, a statistically significant negative correlation was identified between these antibody responses and nodules (*p* < 0.05 in all cases; Table 6). Whilst similar negative relationships were also identified between antigen-specific antibody responses and microfilaridermia, these were not statistically significant (Table S3).

### 3.4. Epitope Mapping Identifies Immunogenic Surface Peptide Sequences of Ov-103 and Ov-RAL-2 

To identify specific epitopes recognised by vaccinated animals, IgG was purified from the sera of a subset of animals at one-week post-booster and 4 months post-exposure (SD62 and SD215, respectively). Antibodies were probed against an array of overlapping *Ov*-103 and *Ov*-RAL-2 peptides representing the full-length proteins, with specific detection of IgG1 and IgG2 isotypes. 

**Figure 5:**
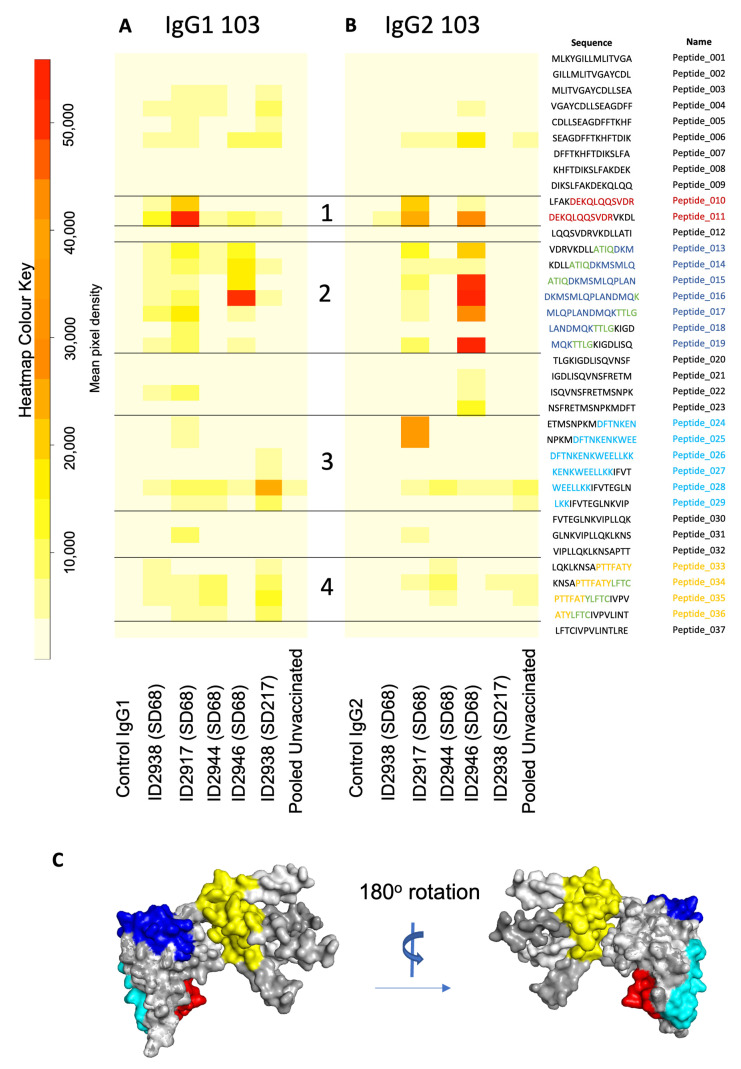
Peptide array heatmaps for IgG purified from IgG of animals vaccinated against *Ov*-103. Whole protein sequence divided into 37 overlapping peptides sequences 15 amino acids in length. Intensity of colour (white to red) shows degree of host antibody recognition (represented by mean pixel density) to (**A**) IgG1 and (**B**) IgG2. IgG controls (provided by JPT, lane 1) and anti-bovine IgG pooled from three unvaccinated, uninfected calves (lane 7) was included on each array, with purified serum IgG from individual vaccinated animals at one-week post-booster (SD62; 2938, 2917, 2944, 2946, lanes 2–5) and a single animal at 4 months post-exposure (SD215; 2938, lane 6). Putative epitopes recognised by IgG from vaccinated animals (1–4) were mapped onto a 3D model of *Ov*-103 using Alphafold2 [36] (**C**); Epitope 1 in red, epitope 2 in navy blue, epitope 3 in cyan and epitope 4 in yellow.

Purified IgG from vaccinated animals exhibited strong recognition of *Ov*-103 peptides (Figure 5A,B, lanes 2–6). Four epitopes were identified corresponding to *Ov*-103 amino acids 41–52 (DEKQLQQSVDR, red), 60–75 (DKMSMLQPLANDMQK, blue), 101–119 (DFTNKENKWEELLKKIFVT, cyan) and 137–152 (PTTFATYLFTCIVPV, yellow) (Figure 5A,B). Epitope 2 showed strong IgG2 antibody recognition across multiple peptides (Figure 5B). Between epitopes 1 and 2, there was no signal detected at peptide 12 despite strong binding of epitopes 11 and 13. The predicted AlphaFold2 3D structure of *Ov*-103 showed that this protein is comprised predominantly of helical structures, with unstructured N-terminal residues 1–14, and C-terminus 135–156. Epitopes 1–3 occur in the folded but solvent accessible surfaces of the protein, whereas epitope 4 is located in the unfolded C-terminal region (Figure 5C).

**Figure 6:**
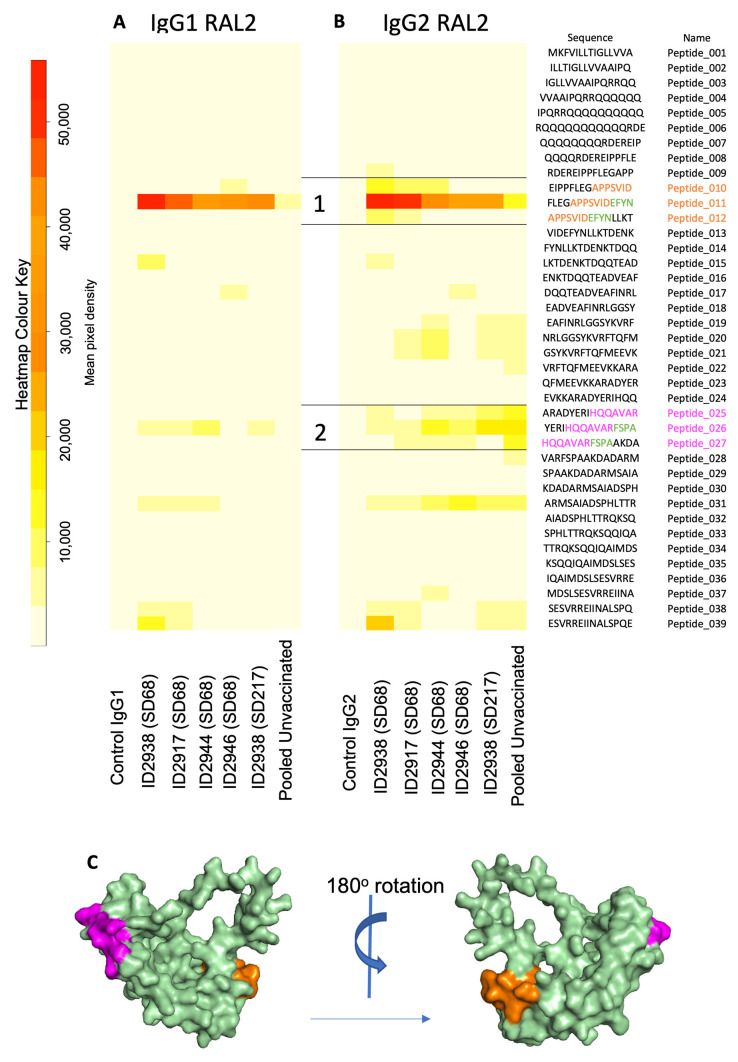
Peptide array heatmaps for IgG purified from IgG of animals vaccinated against *Ov*-RAL-2. Whole protein sequence divided into 39 overlapping peptides sequences 15 amino acids in length. Intensity of colour (white to red) shows degree of host antibody recognition (represented by mean pixel density) to (**A**) IgG1 and (**B**) IgG2. IgG controls (provided by JPT, lane 1) and anti-bovine IgG pooled from three unvaccinated, uninfected calves (lane 7) was included on each array, with purified serum IgG from individual vaccinated animals at one-week post-booster (SD62; 2938, 2917, 2944, 2946, lanes 2–5) and a single animal at 4 months post-exposure (SD215; 2938, lane 6). Putative epitopes recognised by IgG from vaccinated animals (1 and 2) were mapped onto a 3D model of *Ov*-RAL-2 using Alphafold2 [36] (**C**); Epitope 1 in orange and epitope 2 in magenta.

Similarly, purified IgG from vaccinated animals displayed strong recognition of *Ov*-RAL-2 peptides (Figure 6A,B, lanes 2–6). Two epitopes were identified corresponding to *Ov*-RAL-2 amino acids 45–56 (APPSVIDEFYN, orange) and 105–116 (HQQAVARFSPA, magenta). Epitope 1 was strongly recognised by both IgG1 and IgG2 isotype antibodies purified from the sera of vaccinated animals (Figure 6A,B). The AlphaFold 3D model of *Ov*-RAL-2 shows that this protein, like *Ov*-103, is comprised predominantly of helical structures for regions R36-L160, where the per-residue confidence scores (pLDDT) were very high to high; however, the structure of the rest of the protein could not be reliably predicted. The two epitopes mapped onto the folded part of the predicted 3D structure of *Ov*-RAL-2 and also appear to be accessibly located on the protein surface (Figure 6C).

### 3.5. Ov-103 and Ov-RAL-2 Immunisation in Cattle Is Associated with Altered Cellular Immune Responses

In addition to humoral immune responses, as part of the vaccine efficacy trial cellular immune responses were examined through peripheral blood mononuclear cell (PBMC) proliferation assays over the immunisation period, and haematological analysis over the immunisation and exposure period. Multi-variable linear mixed-effect regression analysis identified statistically a significant general increase in total leucocyte, lymphocyte, monocyte, eosinophil and neutrophil counts in both immunised and control animals (*p* < 0.005 in all cases; Table S4), and a statistically significant increase in peripheral monocyte counts in immunised calves relative to control animals over the same period (*p* < 0.05; Table S4). Multi-variable linear mixed-effect regression analysis revealed no statistically significant associations between PBMC proliferation and vaccination status over the immunisation period (SD0–SD83; Table S4). Separate multi-variable linear mixed-effect regression analysis of the exposure period (SD89–SD796) revealed lymphocyte counts increased over the exposure period, whilst monocyte and eosinophil counts decreased independently of immunisation status (*p* < 0.001 in all cases; Table S5).

### 3.6. Calf Health and Diagnostics

Routine diagnostic testing for bovine tuberculosis did not identify infection in any animals over the duration of the study period. Trypanosomosis, piroplasmosis and helminth infections were identified and treated periodically. In the immunogenicity trial, one animal was lost from the Advax-2 group (2905) at SD8, diagnosed with trypanosomosis shortly before death with additional findings of pneumonia and fibrinous pleuritis identified post-mortem; and one animal (2914) died from the alum group at SD95, in which local endemic tick-borne disease (e.g., anaplasmosis) was strongly suspected. In the vaccine efficacy trial, one control animal (2945) died on SD92 after developing haemolytic anaemia, whilst two immunised animals (2928 and 2929) died from lightning strike on SD608. Data for animal 2905 has been excluded from this study, whilst data for the other four animals (2914, 2945, 2928 and 2929) were included up until their final sampling points. 

## 4. Discussion 

The bovine *O. ochengi* natural infection model represents an important opportunity to bridge the gap between highly controlled small animal experimental models and the stochastic nature of human onchocerciasis in terms of both heterogenous populations and host immune responses, as well as natural transmission and exposure. Here, we demonstrate through a vaccine efficacy study that co-administration of recombinant *Ov*-103 and *Ov*-RAL-2 formulated with Montanide^TM^ ISA 206 VG in cattle elicits substantial antigen-specific serum IgG1 and IgG2 responses and a significant reduction in the rate of acquisition of adult and microfilariae following subsequent natural exposure to *O. ochengi*. 

A reduction in the rate of acquisition of both nodules and resulting microfilaridermia in vaccinated animals indicates *O. ochengi* infection and patency established less effectively in vaccinated animals over the study period. This is a highly significant result in the context of developing a human vaccine for onchocerciasis. Based on the differing rates of acquisition identified, a longer exposure period (over multiple years) would result in a wider divergence than the reductions in mean nodule and microfilarial burdens after 24 months (35% and 70%, respectively). This is important, since natural human *O. volvulus* infections occur due to exposure over a timescale of decades. Consequently, such a vaccine would substantially reduce infection rates over a prolonged period and, through reductions in microfilaridermia, limit severity of clinical disease and ongoing transmission. It should be noted, however, that a simple extrapolation of these findings should be carried out with caution, since it is known that density-dependent effects mean neither nodule burdens or microfilarial skin densities accumulate at a constant rate over a host’s lifetime [41]. Nonetheless, these findings may be of considerable value for estimating the potential impact of vaccination and different immunisation strategies on onchocerciasis elimination efforts, such as through the incorporation of observed parameters of vaccine-associated reductions in burdens into existing transmission models [15]. 

In addition to a vaccine-associated reduction in *O. ochengi* burden, the anti-*Ov*-103 and *Ov*-RAL-2-specific IgG1 and IgG2 isotype responses observed in both the immunogenicity and vaccine efficacy trials are of major significance. Specifically, IgG2 responses are of note since in cattle these are stimulated by IFN-γ, and represent an important component of ADCC with substantial expression of IgG2-specific Fc receptors displayed on bovine macrophages, neutrophils and eosinophils [22,23,42]. Furthermore, the observed increase in peripheral blood monocyte counts in vaccinated calves over the immunisation period indicates an antigen-induced innate cellular response. Overall, these results therefore suggest the presence of a protective, balanced Th1/Th2 response to vaccination and subsequent natural challenge. These findings are consistent with a previous experimental vaccine trial in cattle using a multi-antigen vaccine (including recombinant *O. ochengi* RAL-2) formulated in Freund’s complete adjuvant, where reductions in total Mf burdens were accompanied by a strong antigen-specific serum IgG response composed of both IgG1 and IgG2 [21]. A recent study into vaccine-induced protection against *O. volvulus* across multiple outbred strains of mice identified a wide degree of variation in cytokine, humoral and cellular responses across strains, demonstrating the need for onchocerciasis vaccines to induce a broad range of immunological responses in order to maximise its protective effects when administered to heterogenous (human) populations [20]. Collectively, this evidence therefore suggests vaccine formulations for human onchocerciasis should aim to elicit a similarly balanced Th1/Th2 immune response. 

With respect to potential immunological mechanisms associated with vaccine-induced protection, the significant negative correlation identified between antigen-specific (*Ov*-103 and *Ov*-RAL-2) IgG1 and IgG2 antibody titres and adult female worm burdens is highly suggestive of a protective, antibody-dependent vaccine-induced immune response. It is important to acknowledge that, aside from uninformative PBMC proliferation assays, the current study did not consider additional evidence of adaptive cell-mediated immunity and induction of memory T-cells. Further investigation of these responses is planned for ongoing vaccine efficacy trials in cattle. There are numerous prior studies investigating protective immune responses against *O. volvulus*, particularly parasite-specific antibody responses [43,44], and ADCC [45,46]. Evidence of antigen-specific, antibody-dependent protection against *O. volvulus* in humans has been demonstrated previously by use of purified human monospecific anti-*Ov*-103 and *Ov*-RAL-2 antibodies from putatively and concomitantly immune individuals. These inhibited *O. volvulus* L3 moulting when incubated *in vitro* with naïve human monocytes and, in the case of anti-*Ov*-103 antibodies, neutrophils [17]. Notably, monospecific human anti-*Ov*-103 antibodies also inhibited the motility of *O. volvulus* MF in the presence of neutrophils [47]. Moreover, human subjects with putative immunity to *O. volvulus* have been shown to produce parasite-specific, cytophilic antibody responses and exhibit increased IL-5, IFN-γ and GM-CSF production from PBMCs when stimulated with L3, adult female and Mf antigens, relative to patently-infected individuals [48,49,50]. Similarly, sera from *Bm*-103 and *Bm*-RAL-2 vaccinated gerbils have been shown to kill *Brugia malayi* L3 in vitro when incubated with peritoneal exudate cells [16]. Experimental trials in mice immunised with *Ov*-103 and *Ov*-RAL-2 formulated in Advax-2 showed that in addition to the typical Th2-biased cytokine profile induced by exposure to *O. volvulus* and its recombinant antigen candidates, a 2–3 fold increase in IFN-γ was associated with a significant decrease in *O. volvulus* L3 survival in vivo [19].

Both *Ov*-103 and *Ov*-RAL-2 are expressed in the glandular oesophagus of *O. volvulus* L3s, the cuticle and hypodermis of adult female worms, and on the surface of Mf [16,47]. Consequently, protective immune responses raised against these antigens have the potential to impact *O. volvulus* infection at several developmental stages within the host. An anti-*O. volvulus* L3 effect has previously been validated in mice immunised with *Ov*-103 and *Ov*-RAL-2 formulated with both alum and Advax-2 adjuvants [17,19,20,21]; whilst in a gerbil-*Brugia malayi* infection model, immunisation with homologous recombinant *Bm*-103 and *Bm*-RAL-2 antigen homologues formulated in alum yielded a significant reduction in total adult worm burdens and adult female worm fecundity [16]. In the current study, the lower rate of nodule acquisition observed in vaccinated animals (despite nodules being first detected in both experimental groups at a similar time post-exposure) indicates inhibition of development of L3 through to young L5, preventing a proportion of worms from reaching maturity. Similarly, the significant reduction in adult female worm mass in the vaccine group at final timepoint sampling (despite observed nodules being of comparable age to the control group) suggests an ongoing fitness cost to the adult female worm, either through a lasting effect of developmental inhibition, or an ongoing anti-adult female effect. However, this effect did not apparently extend to female worm fecundity, where no significant difference was identified in embryonic development or abnormality between groups. Consequently, if there is an additional vaccine-induced anti-Mf effect, it is likely to manifest after release by the female worm. It should also be noted that whilst patency and presence of live Mf were observed in vaccinated animals, their infectivity of *Simulium* spp. flies relative to Mf produced in control animals was not determined in the current study. Additionally, since natural infection of cattle with *O. ochengi* results in no known negative pathological effects on the host, it is not possible from this study to determine the benefits of this co-administered vaccine from a therapeutic perspective [24]. However, irrespective of whether the observed reduction in Mf burdens is the result of inhibition of larval development, adult female and/or Mf effect(s) or some combination of these, our findings highlight the potential benefits of using these vaccines candidates in terms of an anti-fecundity effect as was observed in experimental *B. malayi* vaccine studies [16,51,52]. In humans, this would help to reduce disease pathology associated with microfilaridermia, and also greatly assist ongoing elimination efforts through lowering of *O. volvulus* transmission. Anti-fecundity effects have been observed as a common and desirable from both a therapeutic and transmission perspective for several other parasitic helminths of human and veterinary importance [53,54,55]. These benefits were previously identified by modelling using the assumption that a vaccine is beneficial if it elicits 50% reduction of adult worm burden and 90% reduction in Mf [15].

The immunogenicity of recombinant *Ov*-103 and *Ov*-RAL-2 has been demonstrated previously on multiple occasions [16,17,19,21,50]. Through analysis of *Ov*-103 and *Ov*-RAL-2 peptide arrays, we have identified that these antigen-specific IgG1 and IgG2 *Ov*-103 and *Ov*-RAL-2 responses are largely restricted to a small number of surface accessible epitopes, with epitope 1 of *Ov*-RAL-2 in particular showing a high degree of response conservation and expression, as well as sustained detection into later-stage infection (SD215). It is notable that there is a partial overlap between the sequence and position of epitope 3 of *Ov*-103 discovered here and that of a previous study where peptide arrays were performed using sera from patently *O. volvulus* infected humans [56]. Furthermore, both epitope 2 and 4 of *Ov*-103 identified in this study have also been identified as both human B-cell and helper T-cell epitope candidates through computational analysis, again suggesting a commonality between bovine and human responses, as well as a potential expansion of their role and importance to stimulation of adaptive T-cell responses [57]. Due to the small sample size, it was not possible to draw any conclusions on the importance of these epitope-specific responses for parasite killing. Nonetheless, these results demonstrate the value of these novel approaches in improving our understanding of host vs. vaccine-induced immune responses from the perspective of ongoing vaccine development. As such, wider investigations of a larger sample set, both within and across species and differing immunological and infection statuses, is likely to be a valuable and worthwhile line of enquiry for further investigation.

Following exposure, the calculated monthly and annual transmission potentials at the transmission site were comparable to those observed commonly in endemic transmission sites for *O. volvulus*; hence, the degree of immunological stimulation and magnitude of host response seen are likely to be comparable to those expected in immunologically naïve human subject over a similar timescale [26]. *Ov*-RAL-2 is known to be immunogenic in people, and has been identified previously as a potential diagnostic marker [58]. However, whilst there was some indication of increase anti-*Ov*-RAL-2 serum IgG response in a small number of vaccinated and control animals post-exposure, these serological responses were modest compared to the vaccine-induced responses observed and were not found to be statistically significant. Given the apparent lack of re-stimulation resulting from natural exposure post-vaccination, there may therefore be a case for reviewing the timing of booster vaccinations to stimulate memory responses and enhance immunological protection post-exposure longer term. To this end, further vaccine efficacy trials in cattle with modification of vaccine regimens are presently ongoing. Similarly, it is possible that following subsidence of the initial systemic responses observed over the immunisation period, an ongoing alteration and/or enhancement of local immune responses to natural exposure and infection may persist. Further investigation of local anti-parasitic inflammatory responses through techniques such as immunohistochemistry and/or *in-situ* hybridisation is therefore indicated. Previous studies investigating both chemotherapeutic and anti-*Wolbachia* treatments have demonstrated the value of similar approaches for characterising the local inflammatory responses to *O. ochengi* nodules [59,60].

Whilst our immunogenicity trial demonstrated both *Ov*-103 and *Ov*-RAL-2 are capable of inducing detectable antigen-specific serum IgG responses with all adjuvants trialled, the water-in-oil-in-water (w/o/w) emulsion Montanide^TM^ ISA 206 VG was clearly the most effective formulation in eliciting a strong, antigen-specific serum IgG1 and IgG2 response and a general increase in peripheral leucocyte counts (including neutrophils and eosinophils) in all study animals over the immunisation period. This indicates a strong, balanced Th1/Th2 response as well as functional B-cell memory, which was reflected by rapid recrudescence of serum antibody responses following booster immunisations. In this study, Advax-2 adjuvanted vaccines induced an IgG1 dominant profile similar to alum, suggesting that the CpG component (which would normally give a strong Th1 bias to the response) was either present at too low a dose or was unable to stimulate bovine TLR-9, its target receptor. TLR-9 ligands can be highly species-specific, with the bovine TLR9 gene having greater sequence homology to human than murine TLR-9, with similar CpG motifs been shown to activate human and bovine but not murine leukocytes [61]. Whilst the CpG in Advax-2 has been shown to be active on mouse and human TLR-9, it has not been tested previously for bovine TLR-9 activity. This will require further investigation. 

The choice of Montanide as adjuvant in the vaccine efficacy trial was justified by the stimulation of innate and cellular responses and the subsequent negative correlation identified between antigen-specific antibody responses and *O. ochengi* female worm burdens, which are supportive of the importance of ADCC mechanisms in vaccine-induced immunological protection. Montanide emulsion adjuvants are proprietary products with a proven track record in veterinary vaccine development with parallels to the current study. In particular, a vaccine trial in cattle using a recombinant antigen candidate formulated in either Montanide ISA 206 VG against the digenean trematode *Fasciola hepatica* demonstrated a significant reduction in adult fluke burdens and fecundity following natural exposure [62]. Importantly, whilst *F. hepatica* infection in cattle is known to modulate immunity towards a non-protective Th2 response dominated by IgG1 antibody responses and a non-proliferative PBMC response [38,63], vaccine and Montanide-induced immunity has been associated with an increase of IgG2 and cellular responses, including polarisation towards classical macrophage activation and function [62,64]. Due to the local granulomatous responses commonly associated with Montanide preparations, these are not commonly used in human vaccine formulations, although there are some notable examples [65,66,67]. However, given the similarly heterogenous population and natural exposure, it is likely human vaccine preparations based on *Ov*-103 and *Ov*-RAL-2 will need to induce a similarly mixed Th1/Th2 response to those observed in the bovine *O. ochengi* model as previously discussed.

Overall, this study strongly demonstrates the potential of recombinant *Ov*-103 and *Ov*-RAL-2 antigen candidates for use as anti-worm burden and anti-fecundity vaccines in the control and elimination of human onchocerciasis. Furthermore, we have determined that in a heterogenous outbred cattle population, immunological protection against natural exposure to *O. ochengi* is associated with a mixed Th1/Th2 antibody-associated response, which will help to inform ongoing pre-clinical and clinical vaccine development, including further trials in cattle and those planned in humans.

## Figures and Tables

**Table 1:** Antigen-specific total IgG, IgG1 and IgG2 antibody titres in immunogenicity trial calves at SD188.

Group	ID	*Ov*-RAL-2			*Ov*-103		
		IgG	IgG1	IgG2	IgG	IgG1	IgG2
Control	2901	5800	0	0	2300	0	0
	2904	0	0	0	0	0	0
	2911	0	0	0	900	0	0
Rehydragel	2906	47,700	15,800	0	19,500	34,200	0
	2908	18,300	15,100	0	9500	47,700	0
Advax-2	2910	12,800	11,900	0	5000	38,800	0
	2912	24,000	28,300	0	8500	44,300	0
Montanide	2907	944,300	669,900	1,086,800	373,900	233,300	27,300
	2909	1,039,700	87,700	868,200	283,200	82,200	721,200
	2913	404,000	524,000	119,900	216,500	183,200	28,200

**Table 2:** Descriptive parasitological statistics for vaccine efficacy trial calves at end-point sampling (SD780–SD794).

	Control Group (*n* = 11)	Vaccine Group (*n =* 10)
Nodule burden	20.2 (±21.0); 3–79	13.2 (±13.6); 2–43
Nodule diameter (mm)	4.8 (0.8); 3.1–7.8 (nodules assessed = 147)	4.7 (0.8); 3.0–7.0 (nodules assessed = 107)
Nodule age * (days)	545 (±89); 293–694 (nodules assessed = 147)	536 (±101); 266–688 (nodules assessed = 107)
Nodule weight (mg)	100.6 (45.6); 22.7–269.4 (nodules assessed = 128)	88.6 (40.3); 24.6–250.1 (nodules assessed = 100)
Male worms recovered per nodule	0.5 (0.7); 0–3 (nodules assessed = 50)	0.7 (1.2); 0–5 (nodules assessed = 38)
*O. ochengi* microfilarial density (per mg skin)	4.7 (±7.9); 0–25.1	1.4 (±1.6); 0–4.5

Values expressed as Mean (±SD); range. * Based on monthly sampling date an individual nodule was first recorded post-exposure.

**Table 3:** Multivariable linear mixed effects regression analysis results for *O. ochengi* infection kinetics in vaccine efficacy trial calves over exposure period (SD89–SD780).

Response Variable (Y)	Fixed-Effect Explanatory Variable (x)	Co-Efficient Value (β)	Standard Error	*p*-Value
Female worm nodules	Time	0.024	0.0017	<0.0001 *
	Sex (m)	2.65	1.72	0.14
	Vaccination:Time *(interaction)*	−0.0062	0.0022	0.0043 *
Microfilarial burden	Time	0.0080	0.0012	<0.0001 *
	Sex (m)	0.76	0.78	0.34
	Vaccination:Time *(interaction)*	−0.0031	0.0012	0.0085 *

Analysis uses residual maximum likelihood variance estimates with individual animal ID as a random-effect explanatory variable. * denotes statistical significance (*p* < 0.05). Results given to 2 decimal places, or 2 significant figures where <0.10.

**Table 4:** Multivariable linear mixed effects regression analysis results for antigen-specific antibody responses in vaccine efficacy trial calves over immunisation period (SD0-SD83).

Anti-*Ov*-103 IgG Responses				
Response Variable (Y)	Fixed-Effect Explanatory Variable (x)	Co-Efficient Value (β)	Standard Error	*p*-Value
Total IgG	Time	−0.00013	0.00038	0.74
	Sex (m)	0.025	0.056	0.66
	Vaccination:Time (*interaction*)	0.0091	0.00051	<0.0001 *
IgG1 titre (logged)	Time	−0.013	0.0091	0.15
	Sex (m)	−0.15	0.43	0.74
	Vaccination:Time (*interaction*)	0.21	0.0090	<0.0001 *
IgG2 titre (logged)	Time	−0.016	0.011	0.17
	Sex (m)	−0.29	0.97	0.77
	Vaccination:Time (*interaction*)	0.16	0.014	<0.0001 *
**Anti-*Ov*-RAL-2 IgG Responses**				
**Response Variable (Y)**	**Fixed-Effect Explanatory Variable (x)**	**Co-Efficient Value (β)**	**Standard Error**	***p*** **-Value**
Total IgG	Time	−0.00020	0.00041	0.62
	Sex (m)	0.039	0.059	0.52
	Vaccination:Time (*interaction*)	0.0083	0.00055	<0.0001 *
IgG1 titre (logged)	Time	−0.018	0.010	0.094
	Sex (m)	0.19	0.49	0.70
	Vaccination:Time (*interaction*)	0.21	0.010	<0.0001 *
IgG2 titre (logged)	Time	−0.0075	0.011	0.50
	Sex (m)	−0.13	0.71	0.85
	Vaccination:Time (*interaction*)	0.15	0.013	<0.0001 *

Analysis uses residual maximum likelihood variance estimates with individual animal ID as a random-effect explanatory variable. * denotes statistical significance (*p* < 0.05). Results given to 2 decimal places, or 2 significant figures where <0.10.

**Table 5:** Multivariable linear mixed effects regression analysis results (2 d.*p*./2 s.f.) for antigen-specific antibody responses in vaccine efficacy trial calves over exposure period (SD89-SD780).

Anti-*Ov*-103 IgG Responses				
Response Variable (Y)	Fixed-Effect Explanatory Variable (x)	Co-Efficient Value (β)	Standard Error	*p*-Value
Total IgG	Time	0.000031	0.000029	0.30
	Vaccination	0.51	0.056	<0.0001 *
	Sex (m)	−0.013	0.058	0.82
	Vaccination:Time (*interaction*)	−0.00062	0.000042	<0.0001 *
IgG1 titre (logged)	Time	0.0040	0.0020	0.053
	Vaccination	12.21	1.17	<0.0001 *
	Sex (m)	0.69	0.87	0.44
	Vaccination:Time (*interaction*)	−0.017	0.0029	<0.0001 *
IgG2 titre (logged)	Time	0.0025	0.0019	0.19
	Vaccination	8.34	1.46	<0.0001 *
	Sex (m)	−0.56	1.34	0.68
	Vaccination:Time (*interaction*)	−0.010	0.0027	0.00054 *
**Anti-*Ov*-RAL-2 IgG Responses**				
**Response Variable (Y)**	**Fixed-Effect Explanatory Variable (x)**	**Co-Efficient Value (β)**	**Standard Error**	***p*** **-Value**
Total IgG	Time	0.000050	0.000031	0.10
	Vaccination	0.43	0.043	<0.0001 *
	Sex (m)	0.047	0.042	0.28
	Vaccination:Time (*interaction*)	−0.00055	0.000044	<0.0001 *
IgG1 titre (logged)	Time	0.0052	0.0017	0.0042 *
	Vaccination	12.68	1.04	<0.0001 *
	Sex (m)	1.51	0.81	0.076
	Vaccination:Time (*interaction*)	−0.016	0.0025	<0.0001 *
IgG2 titre (logged)	Time	0.0039	0.0016	0.020 *
	Vaccination	8.62	1.37	<0.0001 *
	Sex (m)	−0.97	1.31	0.46
	Vaccination:Time (*interaction*)	−0.011	0.0023	<0.0001 *

Analysis uses residual maximum likelihood variance estimates with individual animal ID as a random-effect explanatory variable. * denotes statistical significance (*p* < 0.05). Results given to 2 decimal places, or 2 significant figures where <0.10.

**Table 6:** Multivariable linear mixed effects regression analysis results investigating correlations between female worm nodule burdens (response variable “Y”) and antigen-specific serum IgG responses in vaccinated calves post-exposure nodule antibody responses in vaccine efficacy trial over immunisation period (SD89-SD780).

Anti-*Ov*-103 IgG Responses			
Fixed-Effect Explanatory Variable (x)	Co-Efficient Value (β)	Standard Error	*p*-Value
**Total IgG**	−8.01	1.83	<0.0001 *
Sex (m)	2.62	2.74	0.36
Total IgG:Time *(interaction)*	0.034	0.0059	<0.0001 *
**IgG1 titre (logged)**	−0.52	0.17	0.0059 *
Sex (m)	1.51	1.58	0.36
IgG1 titre (logged):Time *(interaction)*	0.0012	0.00045	0.015 *
**IgG2 titre (logged)**	−0.38	0.18	0.046 *
Sex (m)	1.42	1.75	0.43
IgG2 titre (logged):Time *(interaction)*	0.00095	0.00051	0.073
**Anti-*Ov*-RAL-2 IgG Responses**			
**Fixed-Effect Explanatory Variable (x)**	**Co-Efficient Value (β)**	**Standard Error**	***p*** **-Value**
**Total IgG**	−7.40	1.83	<0.0001 *
Sex (m)	0.42	2.94	0.89
Total IgG:Time *(interaction)*	0.049	0.0050	<0.0001 *
**IgG1 titre (logged)**	−0.55	0.18	0.0057 *
Sex (m)	1.66	1.61	0.33
IgG1 titre (logged):Time *(interaction)*	0.00094	0.00038	0.023 *
**IgG2 titre (logged)**	−0.40	0.19	0.043 *
Sex (m)	1.08	1.75	0.55
IgG2 titre (logged):Time *(interaction)*	0.0011	0.00052	0.044 *

Analysis uses residual maximum likelihood variance estimates with individual animal ID as a random-effect explanatory variable. * denotes statistical significance (*p* < 0.05). Results given to 2 decimal places, or 2 significant figures where <0.10.

## Data Availability

All data used for statistical analyses available from Dryad DOI: https://doi.org/10.5061/dryad.12jm63z0s.

## Supplementary Materials

The following supporting information can be downloaded at: https://www.mdpi.com/article/10.3390/vaccines10060861/s1, Table S1: Calf recruitment and routine health interventions. Figure S1: Confirmation of *O. ochengi* Transmission At Natural Exposure Site. Table S2: Multivariable linear mixed effects regression analysis results for *O. ochengi* infection and parasitological parameters in the vaccine efficacy trial at end-point sampling (SD780–SD794). Table S3: Multivariable linear mixed effects regression analysis investigating correlations between *Onchocerca* spp. microfilaridermia (response variable “Y”) and antigen-specific serum IgG responses in vaccinated calves post-exposure nodule antibody responses in the vaccine efficacy trial over immunisation period (SD89–SD780). Table S4: Multivariable linear mixed effects regression analysis results for cellular immune responses in vaccine efficacy trial calves over immunisation period (SD0–SD83). Table S5; Multivariable linear mixed effects regression analysis results for cellular immune responses in vaccine efficacy trial calves over exposure period (SD89–SD780).
