# Supplementary material for: Co-Administration of Adjuvanted Recombinant Ov-103 and Ov-RAL-2 Vaccines Confer Protection against Natural Challenge in A Bovine Onchocerca ochengi Infection Model of Human Onchocerciasis
Source: Vaccines (Basel). 2022 May 27;10(6):861. doi: 10.3390/vaccines10060861 (PMC9229719; doi:10.3390/vaccines10060861)
Supplement: Supplementary file 1 [file vaccines-10-00861-s001.zip › vaccines-1726194-supplementary.pdf]

Table S1 - Calf recruitment and routine health interventions

Cohort	Animal ID	Sex	Age at SD0 (days)	Experimental group
**Immunogenicity group**	2901	Male	139	Control
	2904	Male	135	Control
	2906	Female	133	Alum
	2907	Female	132	Montanide
	2908	Female	129	Alum
	2909	Female	126	Montanide
	2910	Male	123	Advax-2
	2911	Female	122	Control
	2912	Female	117	Advax-2
	2913	Female	113	Montanide
**Exposure group**	2917	Female	190	Vaccinated
	2918	Female	179	Control
	2921	Male	167	Control
	2922	Male	160	Vaccinated
	2924	Male	132	Control
	2925	Male	129	Control
	2927	Female	110	Control
	2928	Male	111	Vaccinated
	2929	Male	117	Vaccinated
	2930	Male	115	Vaccinated
	2931	Female	124	Vaccinated
	2932	Male	123	Vaccinated
	2933	Female	122	Control
	2934	Male	121	Vaccinated
	2935	Male	120	Control
	2937	Female	119	Control
	2938	Male	118	Vaccinated
	2940	Male	115	Control
	2941	Male	113	Control
	2942	Male	110	Vaccinated
	2943	Male	109	Control
	2944	Male	108	Vaccinated
	2945	Male	105	Control
	2946	Male	103	Vaccinated

Health interventions:

Disease	Intervention	Immunogenicity trial	Exposure trial
Contagious bovine pleuropneumonia	Vaccination	-	Apr 2019, Jul 2020
Lumpy skin disease	Vaccination	-	Apr 2019, Jul 2020
Blackleg (*Clostridium chauvoei*)	Vaccination	-	Jan 2019, Aug 2019, Jan 2020, Sep 2020
Pasteurellosis	Vaccination	-	Jan 2019, Aug 2019, Jan 2020, Sep 2020
Gastrointestinal nematodes	Albendazole 7.5mg/Kg PO[a]	As required	Jan 2019 & as required
Liver flukes	Nitroxynil 10mg/Kg SC[a]	As required	Jan 2019 & as required
Piroplasmosis, Trypanosomosis and Theileriosis	Diminazene aceturate 3.5mg/Kg IM[b]	-	Jan 2019, June 2019, Nov 2019, Apr 2020, Nov 2020

[a] Whole group treatment where infection detected by monthly coprology in >30% of cohort

[b] Whole group treatment strategy supported by routine diagnostic testing to confirm efficacy

Confirmation Of *O. ochengi* Transmission At Natural Exposure Site

Simulium damnosum s.l. biting, infection rates and *O. ochengi* transmission potential were monitored at the natural exposure site by flytrap and dissection. This confirmed natural transmission was occurring, and identified seasonal patterns; Monthly biting rates and transmission potential (MTP) reached a zenith in January 2019 coinciding with exposure of the main study group animals (Figure S1A). Time-series seasonality analysis indicated peak monthly fly infection rates (MIR) and MTP around January in both years (Figure S1 B - C).

Annual biting rates and transmission potential for the complete hydrological year encompassed by the main study exposure period (April 2019 to March 2020) yielded values of 106,390 flies per cow and 7857 *O. ochengi* L3, respectively, with an average L3 infection rate of female nulliparous 7.4% (±3.5% SD) over the same period

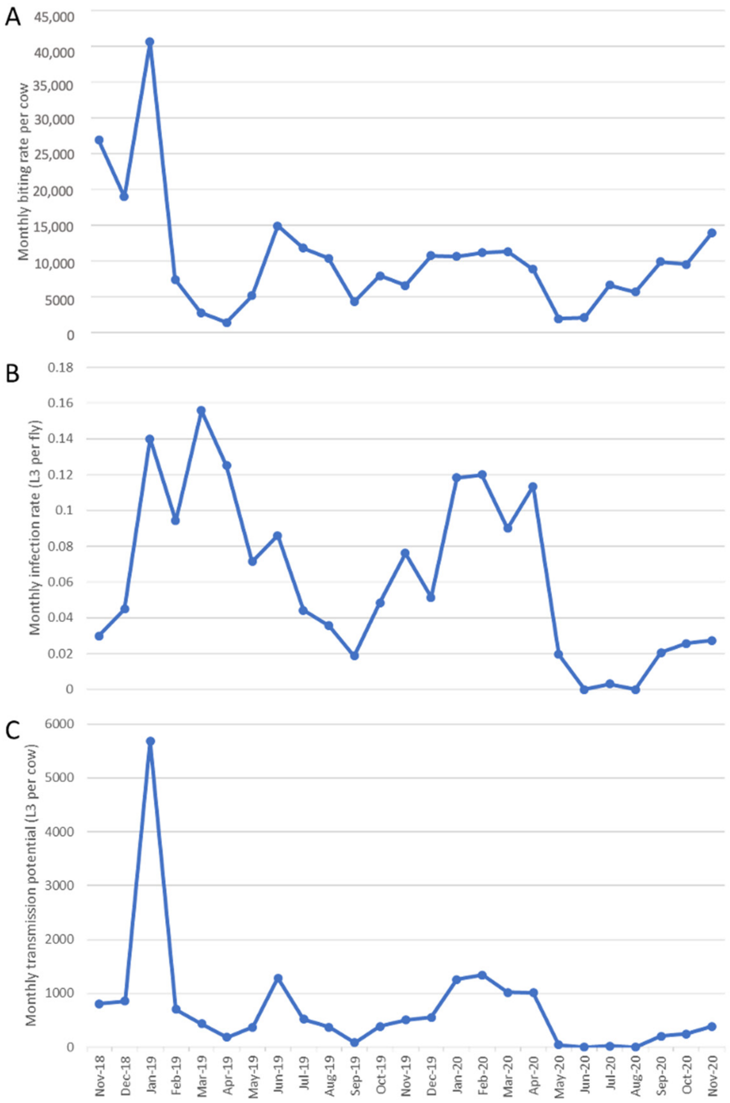

Figure S1. 2018. to November 2020 at the Vina du Sud study site, characterised by ***(A)*** Monthly biting rates as flies per cow, ***(B)*** monthly fly infection rates as number of infective *O. ochengi* L3 larvae per fly and ***(C)*** Monthly transmission rate of infective *O. ochengi* L3 per cow.

Table S2. Multivariable linear mixed effects regression analysis results for *O. ochengi* infection and parasitological parameters in the vaccine efficacy trial at end-point sampling (SD780 – SD794).

Adult worm morphometrics				
**Response variable (Y)**	**Fixed-effect explanatory variable (x)**	**Co-efficient Value (β)**	**Standard Error**	***p*-Value**
Nodule age	Vaccination	4.09	16.65	0.81
	Sex (m)	19.25	19.18	0.33
Average nodule diameter (mm)	Vaccination	0.045	0.19	0.81

	Sex (m)	0.22	0.22	0.32
Nodule weight (mg)	Vaccination	-17.89	8.03	<0.05
	Sex (m)	28.04	9.8	<0.05
Male worms per nodule	Vaccination	0.21	0.23	0.37
	Sex (m)	0.062	0.26	0.81
Female fecundity	Vaccination	-0.22	0.35	0.53
	Sex (m)	0.63	0.4	0.14
Worm age	Vaccination	0.098	0.12	0.41
	Sex (m)	0.1	0.13	0.45
	Worm sex (m)	-0.17	0.06	<0.01
Worm motility	Vaccination	-0.18	0.13	0.19
	Sex (m)	-0.12	0.15	0.41
	Worm sex (m)	0.097	0.048	<0.05
Worm viability (MTT assay OD)	Vaccination	0.015	0.013	0.28
	Sex (m)	0.044	0.015	<0.05
	Worm sex (m)	0.0067	0.012	0.57
**Developmental embryonic stages (count per μL)**				
**Response variable (Y)**	**Fixed-effect explanatory variable (x)**	**Co-efficient Value (β)**	**Standard Error**	***p*-Value**
Total viable embryonic stages	Vaccination	8.04	19.3	0.68
	Sex (m)	34.7	21.16	0.12
Total degenerate embryonic stages	Vaccination	4.2	2.45	0.1
	Sex (m)	−0.75	2.69	0.78
Oocyte count	Vaccination	0.024	1.98	0.99
	Sex (m)	2.72	2.17	0.22
Small morula count	Vaccination	2.47	2.93	0.41
	Sex (m)	4.26	3.2	0.2
Large morula count	Vaccination	1.2	2.61	0.65
	Sex (m)	4.44	2.84	0.13
Pretzel stage count	Vaccination	2.25	8.06	0.78
	Sex (m)	14.11	8.83	0.13
Mature microfilariae	Vaccination	2.09	3.87	0.6
	Sex (m)	6.46	4.24	0.15

Analysis uses residual maximum likelihood variance estimates. * denotes statistical significance ($p < 0.05$). Results given to 2 decimal places, or 2 significant figures where <0.10

Table S3. Multivariable linear mixed effects regression analysis investigating correlations between Onchocerca spp microfilaridermia (response variable "Y") and antigen-specific serum IgG responses in vaccinated calves post-exposure nodule antibody responses in the vaccine efficacy trial over immunisation period (SD89-SD780).

**Anti-*Ov*-103 IgG Responses**			
**Fixed-effect Explanatory Variable (x)**	**Co-efficient Value (β)**	**Standard Error**	***p*-Value**
**Total IgG**	−1.91	1.05	0.07
Sex (m)	0.77	0.82	0.37
Total IgG:Time *(interaction)*	0.0082	0.0029	<0.005
**IgG1 titre (logged)**	−0.024	0.017	0.19
Sex (m)	0.15	0.19	0.45
IgG1 titre (logged):Time *(interaction)*	0.000056	0.000042	0.22
**IgG2 titre (logged)**	−0.021	0.018	0.27
Sex (m)	0.17	0.19	0.39
IgG2 titre (logged):Time *(interaction)*	0.000073	0.000043	0.12
**anti-*Ov*-RAL2 IgG responses**			
**Fixed-effect Vxplanatory Variable (x)**	**Co-efficient Value (β)**	**Standard Error**	***p*-Value**
**Total IgG**	−1.86	1.14	0.1
Sex (m)	0.4	0.85	0.65
Total IgG:Time *(interaction)*	0.00726	0.0028	<0.05
**IgG1 titre (logged)**	−0.02771	0.0178	0.16
Sex (m)	0.15	0.19	0.44
IgG1 titre (logged):Time *(interaction)*	0.00004	0.00004	0.33
**IgG2 titre (logged)**	−0.03124	0.01815	0.12
Sex (m)	0.12	0.18	0.52
IgG2 titre (logged):Time *(interaction)*	−0.00001	0.00004	0.79

Analysis uses residual maximum likelihood variance estimates with individual animal ID as a random-effect explanatory variable. * denotes statistical significance ($p < 0.05$). Results given to 2 decimal places, or 2 significant figures where <0.10

Table S4. Multivariable linear mixed effects regression analysis results for cellular immune responses in vaccine efficacy trial calves over immunisation period (SD0-SD83).

**PBMC Stimulation Index**				
**Response Variable (Y)**	**Fixed-effect Explanatory Variable (x)**	**Co-efficient Value (β)**	**Standard Error**	***p*-Value**
ConA	Time	−0.0037	0.0048	0.44
	Sex (m)	0.052	0.25	0.84
	Vaccination:Time *(interaction)*	0.0019	0.0054	0.73
Ov-103	Time	−0.0020	0.0013	0.14
	Sex (m)	0.039	0.063	0.54
	Vaccination:Time *(interaction)*	0.0015	0.0014	0.30
Ov-RAL2	Time	−0.0036	0.0016	<0.05 *
	Sex (m)	0.018	0.074	0.81
	Vaccination:Time *(interaction)*	0.0026	0.0017	0.13
**Peripheral Blood Leucocyte Counts Per mL**				
**Response Variable (Y)**	**Fixed-effect Explanatory variable (x)**	**Co-efficient Value (β)**	**Standard Error**	***p*-Value**
Total leucocytes	Time	24,310.85	2001.06	<0.0001 *
	Sex (m)	141,205.60	204,054.80	0.50
	Vaccination:Time *(interaction)*	2423.05	2671.86	0.37
Lymphocytes	Time	16,403.98	1460.80	<0.0001 *
	Sex (m)	133,201.60	128,483.90	0.31
	Vaccination:Time *(interaction)*	333.04	1910.60	0.86
Monocytes	Time	508.84	177.84	<0.005 *
	Sex (m)	5895.42	7662.80	0.45

	Vaccination:Time *(interaction)*	367.08	181.86	0.046
Eosinophils	Time	2092.41	423.13	<0.0001 *
	Sex (m)	−31576.50	25,248.43	0.22
	Vaccination:Time *(interaction)*	655.62	502.85	0.19
Neutrophils	Time	5470.17	1173.99	<0.0001 *
	Sex (m)	34796.04	80,303.44	0.67
	Vaccination:Time *(interaction)*	618.46	1455.48	0.67

Analysis uses residual maximum likelihood variance estimates with individual animal ID as a random-effect explanatory variable. * denotes statistical significance ($p < 0.05$). Results given to 2 decimal places, or 2 significant figures where <0.10

Table S5. Multivariable linear mixed effects regression analysis results for cellular immune responses in vaccine efficacy trial calves over exposure period (SD89-SD780).

**Peripheral blood leucocyte counts per mL**				
**Response Variable (Y)**	**Fixed-effect explanatory Variable (x)**	**Co-efficient Value (β)**	**Standard Error**	***p*-Value**
Total leucocytes	Time	298.12	213.91	0.16
	Vaccination	−224,476.00	159,526.00	0.17
	Sex (m)	−88824.70	94,196.88	0.36
	Vaccination:Time *(interaction)*	132.80	304.19	0.66
Lymphocytes	Time	766.14	165.69	<0.0001 *
	Vaccination	−100,973.00	119,698.20	0.41
	Sex (m)	−74,129.6	64,777.94	0.27
	Vaccination:Time *(interaction)*	3.76	235.40	0.99
Monocytes	Time	−190.42	21.68	<0.0001 *
	Vaccination	−22,418.80	14,988.87	0.15
	Sex (m)	3056.35	6875.83	0.66
	Vaccination:Time *(interaction)*	23.60	30.73	0.44
Eosinophils	Time	−108.40	24.23	<0.0001 *
	Vaccination	1821.20	16,784.13	0.91
	Sex (m)	−21,514.50	7758.45	<0.05 *
	Vaccination:Time *(interaction)*	−6.91	34.36	0.84
Neutrophils	Time	−109.68	67.99	0.11
	Vaccination	−217,703.00	165,865.80	0.20
	Sex (m)	115,740.80	176,155.60	0.52
	Vaccination:Time *(interaction)*	38.72	96.88	0.69

Analysis uses residual maximum likelihood variance estimates with individual animal ID as a random-effect explanatory variable. * denotes statistical significance ($p < 0.05$). Results given to 2 decimal places, or 2 significant figures where <0.10
